# Fusions of Consciousness

**DOI:** 10.3390/e25010129

**Published:** 2023-01-09

**Authors:** Donald D. Hoffman, Chetan Prakash, Robert Prentner

**Affiliations:** 1 Department of Cognitive Sciences, University of California, Irvine, CA 92697, USA; 2 Department of Mathematics, California State University, San Bernadino, CA 92407, USA; 3 Association for Mathematical Consciousness Science, D-80539 Munich, Germany; 4 Munich Center for Mathematical Philosophy, LMU Munich, D-80539 Munich, Germany

**Keywords:** consciousness, qualia, subjective experience, hard problem of consciousness, panpsychism, combination problem, conscious agents, interface theory of perception, decorated permutations, amplituhedron, positive Grassmannian, Markov chains, Markov polytopes, fusion simplex

## Abstract

What are conscious experiences? Can they combine to form new experiences? What are conscious subjects? Can they combine to form new subjects? Most attempts to answer these questions assume that spacetime, and some of its particles, are fundamental. However, physicists tell us that spacetime cannot be fundamental. Spacetime, they say, is doomed. We heed the physicists, and drop the assumption that spacetime is fundamental. We assume instead that subjects and experiences are entities beyond spacetime, not within spacetime. We make this precise in a mathematical theory of *conscious agents*, whose dynamics are described by Markov chains. We show how (1) agents combine into more complex agents, (2) agents fuse into simpler agents, and (3) qualia fuse to create new qualia. The possible dynamics of *n* agents form an n(n−1)-dimensional polytope with $n^{n}$ vertices—the *Markov polytope* $M_{n}$. The total fusions of *n* agents and qualia form an (n−1)-dimensional simplex—the *fusion simplex* $F_{n}$. To project the Markovian dynamics of conscious agents onto scattering processes in spacetime, we define a new map from Markov chains to decorated permutations. Such permutations—along with helicities, or masses and spins—invariantly encode all physical information used to compute scattering amplitudes. We propose that spacetime and scattering processes are a data structure that codes for interactions of conscious agents: a particle in spacetime is a projection of the Markovian dynamics of a communicating class of conscious agents.

## 1. Introduction

Consciousness is perplexing, even for expert researchers. Witness the recent plethora of conflicting theories [1]. Even their core ideas are at odds: quantum states of neuronal microtubules [2,3,4], causal architectures that integrate information [5,6,7,8], neuronal global workspaces [9,10,11,12], user illusions and attentional schemas [13,14,15], panpsychism [16,17], and various forms of dualism [18,19]. However, most of these theories of consciousness agree on a key assumption: spacetime, and some of its particles, are fundamental, i.e., ontologically primitive, irreducible, and non-emergent. For example, physicalist theories assume this and nothing more, while many panpsychists likewise assume this but would add that the “intrinsic nature” [20] of such particles is nothing other than consciousness.

It is natural to assume that spacetime is fundamental. Indeed, one might argue that any theory must assume this to respect some variety of naturalism [21]. So why bother to point it out? Because spacetime and its particles are doomed, as a fundamental posit in any theory of reality. Current research in physics tells us so. We sketch, in Section 2, how the interaction of gravity and quantum theory leads physicists to this stunning conclusion.

Remarkably, evolution by natural selection agrees: spacetime and objects are not fundamental. We explain, in Section 3, how evolutionary games reveal that our perceptions of space, time, and objects are no more than a user interface that guides adaptive action.

An interface? To what? Both physics and evolutionary theory are silent. We propose, in Section 4, that conscious agents are fundamental. Some agents interact with others via an interface whose format is spacetime and objects located in spacetime. We offer a precise definition of conscious agent.

When agents interact, what happens? We demonstrate, in Section 5, that they can combine into a complex agent, or fuse into a simpler agent with a novel conscious experience. If *n* agents interact, their possible combinations form an n(n−1)-dimensional polytope with $n^{n}$ vertices—the *Markov polytope* $M_{n}$. Their possible fusions form an (n−1)-dimensional simplex—the *fusion simplex* $F_{n}$.

If agents are fundamental and spacetime is their interface, how precisely do agents create a spacetime interface? As it happens, theoretical physicists have recently peered beyond spacetime and discovered new structures beyond spacetime, such as the amplituhedron [22] and cosmological polytope [23]. They generate spacetime and quantum theory by projection. The essence of these structures, their invariant physical content, can be derived from what is known as “decorated permutations” (in non-supersymmetric theories helicities, or masses and spins, are also required [24]). This is discussed briefly in Section 6 but leaves open the question: what beyond spacetime is permuted, and why? We submit, in Section 7, that decorated permutations are a convenient précis of the dynamics of conscious agents, and we conjecture how to relate spacetime-physics to the combination and fusion of conscious agents.

Agents are no ephemerals in spacetime; spacetime is a data structure for compact representation of agent dynamics. For this reason, spacetime is not fundamental. It is an interface. However, if spacetime is an interface, then its objects, such as neurons and brains, are icons in the interface—useful fictions. Neurons have no causal powers. Standard interpretations for neural correlates of consciousness assume otherwise. We discuss, in Section 8, how these interpretations must be revised. We conclude by noting limitations of the theory of conscious agents, and scouting new directions for its development.

## 2. Spacetime Is Doomed

Most theories of consciousness take spacetime as fundamental. Most theoretical physicists do not. The disconnect is striking, and is a strike against most theories of consciousness.

Physicists tell us that spacetime lacks operational meaning for distances smaller than the Planck length, roughly $10^{-33}$ centimeters, or durations shorter than the Planck time, roughly $10^{-43}$ s [25]. They recognize that “classical spacetime is not a fundamental ingredient of the world, but a construction consisting of more fundamental degrees of freedom” [26]. This view is not exotic. On the contrary, many physicists believe this, no matter what particular approach to spacetime they prefer. However, despite this consensus that spacetime is not fundamental, most theories of consciousness in cognitive neuroscience, artificial intelligence, and philosophy of mind still include spacetime among their foundational entities. In this section, we review some arguments from physics for the doom of spacetime [25]. There are many more, but for the sake of our discussion we will limit ourselves to the following two.

The first argument starts with a simple fact: to measure smaller objects we need light, or other radiation, with shorter wavelengths. Quantum theory tells us that if the wavelength, λ, of the radiation decreases then its energy, *E*, increases,

(1) $$E=\frac{hc}{\lambda }$$

where *c* is the speed of light and *h* is Planck’s constant. This is why particle colliders, such as the Large Hadron Collider, use higher energies to probe smaller particles.

Without gravity, there would be no obstruction. We could in principle generate endlessly higher energies to probe ever smaller particles. However, gravity spoils the party. Einstein taught us that mass and energy are equivalent,

(2) $$E=mc^{2},$$

where *m* is mass. Einstein also taught us that mass curves spacetime, with greater mass creating greater curvature. So, as the wavelength of the radiation shrinks, the concentration of mass-energy grows, creating greater curvature in spacetime. As the wavelength nears the Planck scale, the curvature of spacetime becomes so great that radiation cannot escape. A black hole is born, and destroys the object we want to observe. If we persist, and use even higher energies, the black hole just becomes bigger. Thus, no operational meaning can be given to distances and durations below the Planck scale.

A second argument turns on the quantum theory of measurement. Suppose we have a room of fixed size that contains a measuring device and a particle to be measured. Every measuring device is a physical system. As such, it is subject to the quantum uncertainty for energy and time, and to the uncertainty for position, *x*, and momentum, *p*,

(3) $$\begin{array}{cc} \Delta E\Delta t & \geq h/(4\pi ), \end{array}$$

(4) $$\begin{array}{cc} \Delta x\Delta p & \geq h/(4\pi ). \end{array}$$

The uncertainty principle dictates that to make the device more accurate we must add degrees of freedom. As we add them, we add more parts to the device, and it becomes more massive. Eventually, the device collapses into a black hole and destroys the measurement.

Arguments like these have prompted many physicists to conclude that spacetime is not fundamental. David Gross, for instance, says “I believe that space for sure, and probably time as well, will be emergent” [25]. In this same paper, Gross quotes Ed Witten saying, “Space and time may be doomed”; Nathan Seiberg, “I am almost certain that space and time are illusions”; and Andrew Strominger, “The notion of spacetime is clearly something we’re going to have to give up.” In his 2010 Cornell Messenger Lecture [27], Nima Arkani-Hamed says, “the very notion of spacetime is not a fundamental one. Spacetime is doomed. There is no such thing as spacetime fundamentally in the actual underlying description of the laws of physics.” Arkani-Hamed [28] also argues that quantum theory itself is not fundamental, and will arise with spacetime from some deeper structure: “So there’s some other structure that we’re looking for, and some way of thinking about interpreting this structure will let us see spacetime and quantum mechanics emerge simultaneously and joined at the hip.”

If spacetime is not fundamental, neither are its particles, which are irreducible representations of the Poincaré symmetries of spacetime. Nor are macroscopic objects, such as neurons and brains, made of particles. A theory of consciousness that starts with spacetime, particles, neurons, or brains has little chance. This is particularly problematic for versions of panpsychism that assume spacetime is the stage where the drama of consciousness plays out [29].

## 3. Evolutionary Games

Quantum field theory and gravity together tell us that spacetime and objects in spacetime are doomed. However, how is this consistent with another pillar of modern science, the theory of evolution by natural selection? Evolution describes how certain objects called organisms evolve in time and forage for resources distributed in space.

Usually, this is taken to imply that evolution by natural selection provides us with a grip on the true structure of reality. Surely organisms that see reality more accurately are necessarily more fit—more likely to pass on their genes. We are here today because our ancestors saw reality more accurately than their competition. For example, according to the philosopher and psycholinguist Jerry Fodor, “there is nothing in the ’evolutionary’, or the ’biological’, or the ’scientific’ worldview that shows, or even suggests, that the proper function of cognition is other than the fixation of true beliefs” [30].

It may seem obvious that perceiving the truth must make you more fit. However, this is false, and flatly contradicted by the mathematics of evolution itself. As the philosopher Patricia Churchland puts it, “[t]he principle chore of brains is to get the body parts where they should be in order that the organism may survive. Improvements in sensorimotor control confer an evolutionary advantage: a fancier style of representing [the world] is advantageous so long as it is geared to the organism’s way of life and enhances an organism’s chances for survival. Truth, whatever that is, takes the hindmost” [31].

The cognitive scientist Steven Pinker agrees: “Our minds evolved by natural selection to solve problems that were life-and-death matters to our ancestors, not to commune with correctness” [32]. Pinker scouts several counterexamples to the idea that perceiving truth enhances fitness [33]. For instance, computing the truth takes valuable resources, such as time and energy; this can favor the evolution of heuristics that cut corners on truth and resources. The desire for social acceptance can lead one to adopt false beliefs as a form of virtue signaling. Strategic lies can be advantageous; the best liars are those who are not aware they are lying.

Pinker goes on, however, to admit that, “The idea that the mind is designed for truth is not completely wrong. We do have some reliable notions about the distribution of middle-sized objects around us …” [33] Churchland agrees. She assumes that certain middle-sized objects, such as brains and body parts, are not fictions.

However, middle-sized objects, it turns out, are not exempt. This becomes clear once one distinguishes between informal accounts of biological evolution and mathematical treatments based on evolutionary game theory. It is only the former that appear to assume the reality of spacetime and middle-sized objects.

Simulations and theorems using the tools of evolutionary game theory reveal a counterintuitive result: the probability is zero that any sensory system has ever been shaped to report any true structures of objective reality [34,35,36]. Our senses do not show us truths about objective reality. They simply guide adaptive action.

A key insight from these studies centers on the “fitness payoffs” that govern evolution. One can think of evolution as like a video game in which one must collect points and avoid death to reach the next level of the game. Fitness payoffs in evolutionary theory are like those points. Organisms that reap more fitness payoffs send their genes to the next generation.

Fitness payoffs thus shape the evolution of sensory systems. Are the senses shaped to report truths about the structures of objective reality? No, for the simple reason that fitness payoff functions are, almost surely, not homomorphisms of the structures of objective reality [37]. That is, fitness payoffs lack information about the structures of objective reality, and hence cannot shape the senses to perceive those structures.

However, does this argument not refute itself? It uses math and logic to prove that human cognitive capacities, such as math and logic, are unreliable. Not at all. The arguments here only target the evolution of sensory systems, not the evolution of all cognitive capacities. Each capacity must be studied separately. In the case of math and logic, there are selection pressures for some proficiency: we can reap fitness advantages by accurate reasoning about fitness payoffs. For instance, two bites of a pear offer more fitness payoffs than one bite. The pear is not objective reality; it is a representation of fitness payoffs that can occur for different actions, such as biting it. Reasoning accurately about these fitness payoffs can enhance fitness, even if our sensory systems do not reveal objective reality.

If our senses are not a window on reality, then what are they? A useful metaphor is to think of our senses as providing a user interface to an unknown reality, much like the desktop interface on a laptop ([34,38,39,40,41,42,43,44,45,46,47]; see also [48,49,50] for von Uexküll’s related idea of an Umwelt). The icons on the desktop let you control voltages in circuits of the laptop without you having to know anything about these circuits. If you had to toggle voltages to craft an email, no one would hear from you. Knowing the truth will not make you more fit; it will make you extinct. Evolution shaped our senses to guide adaptive action, not to know the truth. This is called the “*interface theory of perception*” (ITP).

So, evolution by natural selection agrees with physics that spacetime and objects, as reported by our senses, are not fundamental reality. They are simply data structures that guide adaptive action [51], i.e., that help us reap fitness payoffs. There are countless fitness payoff functions relevant to our actions. We hierarchically cluster these payoff functions and compress the clusters into convenient units that we call macroscopic objects. The actions and payoffs appropriate to a pear are quite different from those appropriate to poison ivy. The tens of thousands of perceptual units that we call objects are the way our senses handle the plethora of fitness payoffs that we must deal with. We do not deal with this plethora by seeing the truth. We deal with it by creating thousands of objects as compact data structures that guide context-appropriate actions.

This sensory process of clustering fitness payoffs into objects can be “cognitively impenetrable”: its inner workings can proceed independently of higher cognitive states, such as goals and beliefs. Moreover, the construction of objects can occur simultaneously in multiple sensory systems, such as vision and touch; this creates concurrent mappings from a single stimulus to multiple perceived objects, such as a visual kitten and a tactile kitten that are perceived simultaneously when one sees and pets a kitten. (For some people, the simultaneous visual object and tactile object are not even recognized as arising from the same stimulus [52]). One’s cognitive goals can guide attention among the objects delivered by a sensory system, and this attention can trigger the sensory system to provide more details on an object of interest. This mapping of a single stimulus into distinct objects in concurrent sensory systems contradicts a recent claim: “Critically, as an implementation of cognitive impenetrability, the agent must always use the same mapping from stimulus (resource) to percept (color), regardless of what the current payoff function is” [53]. This mistaken claim has been used to justify a further mistaken claim that sensory systems, if forced to handle many payoff functions, will be shaped by natural selection to construct veridical perceptions [53].

Objects are not mind-independent entities. They are data structures that we construct and employ as needed, and then garbage-collect when, for the moment, we no longer need them. We create each object with a glance and delete it with a blink, or a glance away. Objects do not exist when they are not observed. Thus, they have no definite values of physical properties, such as position or momentum when they are not observed. This aligns with the conclusion of quantum physics that local realism and non-contextual realism are both false [54,55,56].

However, what about, say, a high-speed train bulleting down a track? If you think it is not there when you do not look, why not step in front of it and simply look away?

The answer is that evolution shaped our senses to guide adaptive behavior. We must take them seriously. However, that does not entail that we are entitled to take them literally. To compare, I must take a blue, rectangular icon on my desktop seriously. If I drag it to the trash, I could lose my file. However, the file is not literally blue or rectangular. Similarly, when I see a train I am interacting with some reality that I must take seriously, and that reality exists whether I look or not. However, that reality is not a train. Indeed, as we will discuss, that reality is beyond space and time.

This applies to neurons and brains. They do not exist when they are not observed. We create them when we look and delete them when we look away. Thus, neurons create none of our behaviors or conscious experiences. Theories of consciousness that assume otherwise contradict the clear implications of physics and evolution.

However, is this not too hasty? Do we not have good evidence that brains exist and cause our conscious experiences? Consider the long list of neural correlates of consciousness that have been found. Activity in area V4 of the visual cortex, for instance, is correlated with our conscious experience of color. If a transcranial magnetic stimulation (TMS) device is used to inhibit area V4 in the left hemisphere of the brain of an awake person, then that person will lose all color experience in their right visual field. Everything looks like a black and white photograph. If the TMS device is removed, then the person will see color flow back into their right visual field. Intervening on the brain causes changes in conscious experiences. So, apparently, brains must exist and cause our conscious experiences. As Gerald Edelman put it, “There is now a vast amount of empirical evidence to support the idea that consciousness emerges from the organization and operation of the brain … The question then becomes: What features of the body and brain are necessary and sufficient for consciousness to appear?” [57].

However, this conclusion does not follow. Consider a virtual-reality tennis game. I hit a virtual tennis ball with my virtual tennis racket. I intervene with my virtual racket, and it causes the virtual ball to move. However, this does not entail that the virtual racket exists when I do not perceive it. In fact, the racket does not exist when it is not perceived. Additionally, it has no causal powers. In this metaphor, what has causal powers is some complicated supercomputer, and what I am really doing is toggling millions of voltages in that computer. If I had to toggle them explicitly, I would be overwhelmed. The racket is just a useful fiction that lets me play the game. The same is true of physical objects, such as neurons.

If neurons do not exist when they are not perceived, if they are just useful fictions, should the science of consciousness ignore neuroscience? Not at all. On the contrary, we need more funding for neuroscience, not less. The relationship between neural activity and conscious experience is far more complex than is usually imagined. We need to reverse engineer neurons to understand the deeper reality behind the useful fiction.

We can think of ITP in terms of a distinction that some philosophers have drawn between primary and secondary qualities. John Locke argued that “primary qualities” of objects, such as their “bulk, figure, or motion” exist when unperceived, but that “secondary qualities” of objects, such as their “colors and smells” do not. He then claimed that “... the ideas of primary qualities of bodies are resemblances of them, and their patterns do really exist in the bodies themselves, but the ideas produced in us by these secondary qualities have no resemblance of them at all” [58]. ITP says that *all* qualities are secondary qualities. ITP thus agrees with Immanuel Kant who proposed to “go farther, and for weighty reasons rank as mere appearances the remaining qualities of bodies also, which are called primary, such as extension, place, and in general space” [59].

## 4. Conscious Agents

Quantum theory and gravity tell us that spacetime is doomed. Spacetime and objects are not fundamental reality. Evolution by natural selection agrees.

What, then, is fundamental? These theories are silent. Our theory of spacetime tells us the limits of spacetime, but it cannot reveal what lies beyond. Our theory of evolution tells us that our sensory systems, which show us objects in space and time, are not a window on truth. They are a user interface. However, an interface to what? What reality lies beyond our spacetime interface? Evolution cannot say.

So, we must take a leap. We must propose some reality beyond spacetime. We must then propose precisely how this deeper reality maps onto spacetime. Additionally, we must show that this deeper reality looks like quantum field theory and evolution by natural selection when it is projected onto spacetime.

We propose that consciousness is fundamental, and can be modeled as a network of interacting “conscious agents”. In this section, we briefly motivate and present a definition of conscious agents. A more detailed development is presented elsewhere [60,61].

Here, we seek minimal posits about consciousness that permit the construction of a general theory. This makes our approach unusual. Most scientific theories of consciousness posit physical systems, or functional properties of physical systems. Some propose that these give rise to consciousness; for instance, global workspace [10,62,63], integrated information [8,64], and orchestrated objective reduction [3]. Others propose that these give rise to the illusion of consciousness; for instance, illusionism [14] and attention schema [15]. All of the above falsely assume that spacetime physics is fundamental. This is not the case, for example, in the system of G.W. Leibniz [65] who proposed that simple substances (“monads”) are the ultimate constituents of the universe and that physics, as it was known back then, would result from the dynamics of a network of such monads (Other related views, sometimes referred to as “objective idealism”, are discussed in [66]) Our own view has some similarities with Leibniz’, but also some differences.

We start with two posits about consciousness: (1) there are conscious experiences; and (2) there are probabilistic relations among conscious experiences. These posits lead us to the notion of a conscious agent. Our posits for the notion of a conscious agent mirror G.W. Leibniz’s posits for his notion of a simple substance: “there is nothing besides perceptions and their changes to be found in the simple substance. Additionally, it is in these alone that all the internal activities of the simple substance can consist” [65]. Leibniz further introduced the notion of “appetitions” to describe the monad’s capacity to bring about changes in its (internal) state. In the theory of conscious agents, this is mirrored by the decisions an agent could take. Leibniz believed that any monad perceptually mirrors the whole universe. We slightly adapt Leibniz view here by introducing actions an agent could take on the world. In turn, the world could “perceive” the agent, analogously as the agent perceives its world.

**Figure 1:**
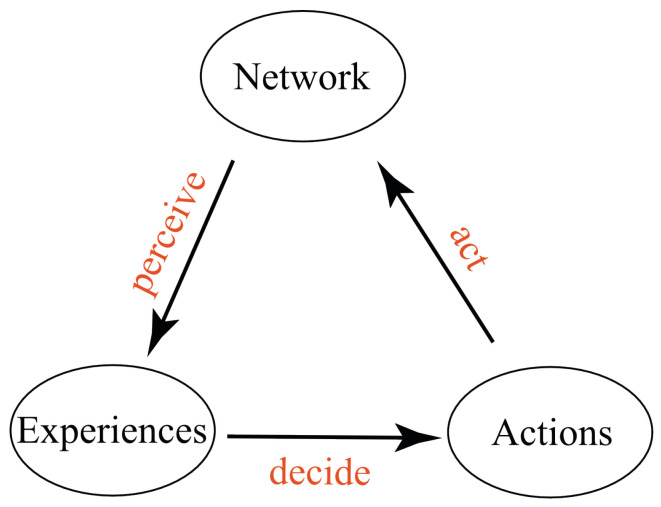
Informal picture of a conscious agent, who acts on a network of other agents, based on its experiences.

Informally, as shown in Figure 1, a conscious agent has a set of possible experiences. It also has a set of possible actions. It is embedded in a world, which we assume to be a network of conscious agents (the thesis of “conscious realism”, [60]). Based on its current experience, the conscious agent decides what action to take. It then acts to affect the experiences of conscious agents in the network. It then perceives a new experience, influenced by the network.

We formalize these posits using (1) measurable spaces for conscious experiences and (2) Markovian kernels for probabilistic relations among conscious experiences. Recall that a measurable space, (X,X), specifies the elementary outcomes, *X*, and possible events, X, for a probabilistic experiment. If, for instance, the experiment is one toss of a coin, then the elementary outcomes are X={H,T}, where *H* denotes “heads” and *T* denotes “tails”. The possible events are X={{H},{T},X,∅}. The set of events, X, is a σ-algebra: in standard probability theory it is closed under complement and countable union, and thus also under countable intersection. A set of elementary outcomes can have more than one σ-algebra. In our coin-toss example, for instance, one could let X={X,∅} (either we observe anything or nothing at all), but not X={{H},∅} (not closed under complement) or X={{H},{T},∅} (not closed under countable union).

With these tools, we can construct a precise definition of conscious agent. A conscious agent, *C*, has a set of potential conscious experiences, *X*, that form a measurable space. It has a measurable space of potential actions, *G*, that it may take in response to its experiences. Its actions influence the experiences of a network of conscious agents. We assume the network as a whole includes the agent in question and has a well-defined set *W* of states, which itself forms a measurable space. In the following, we will refer to this set *W*, of states of the world network, simply as “the world”.

Additionally, recall that a Markovian kernel can be represented, in the finite case, as a matrix in which (1) all entries are non-negative and (2) the entries in each row sum to 1. For instance, a kernel, *K*, relating two experiences to three actions would be a 2×3 matrix, such as

(5) $$K=\left[\begin{array}{ccc} .1 & .3 & .6\\ .4 & .4 & .2 \end{array}\right].$$

When agent *C* has a particular conscious experience x∈X, it chooses an action g∈G. This choice is described by a Markovian kernel, *D*, the “decision kernel”. When a particular action g∈G is taken, it influences the experiences of the agents in the network *W*. This influence is described by a Markovian kernel, *A*, the “action kernel”. The network, *W*, of conscious agents in turn influences the conscious experiences of *C* via a Markovian kernel, *P*, the “perception kernel”.

**Figure 2:**
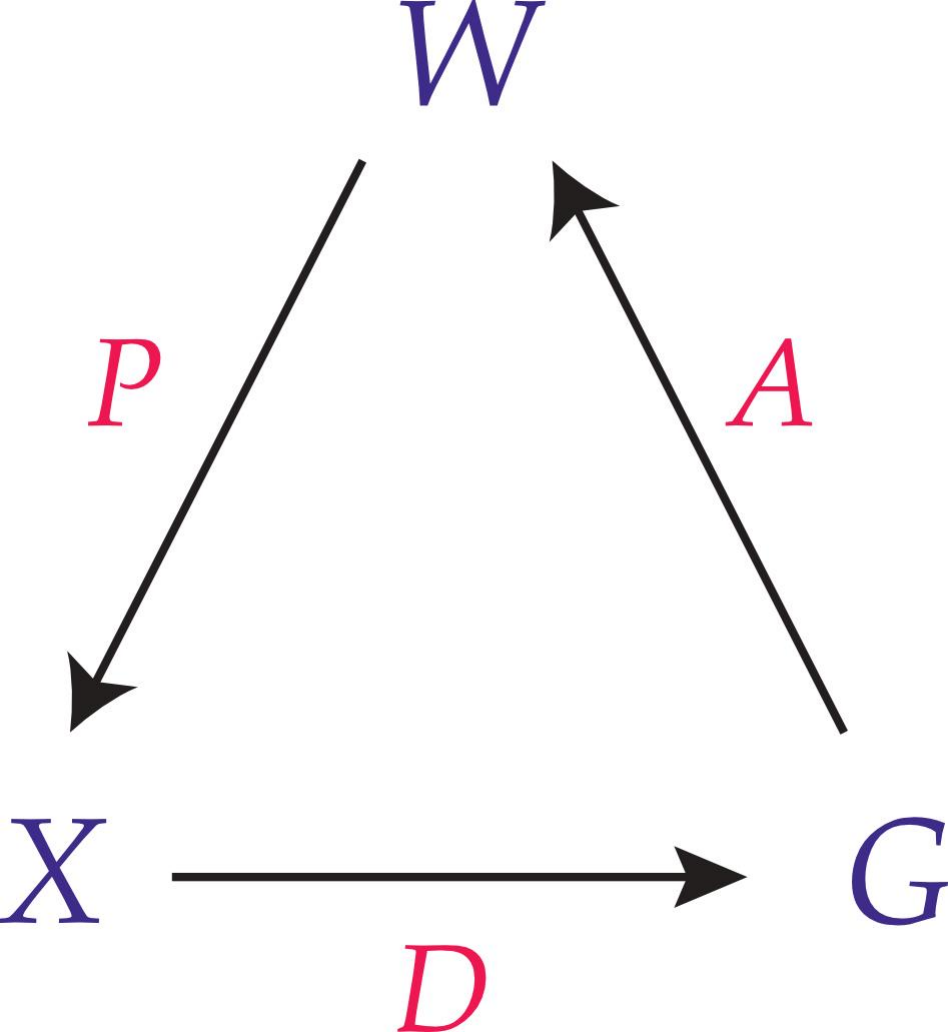
Conscious agent diagram.

Formally, a conscious agent, *C*, is a 6-tuple:

(6) $$C=\left((X,X),(G,G),(W,W),P,D,A\right),$$

where X,G,W are σ-algebras on the sets X,G, and *W*, respectively, so that (X,X),(G,G),(W,W) are measurable spaces, and

(7) $$\begin{array}{cc} P:W\times X & \to \left[0,1\right], \end{array}$$

(8) $$\begin{array}{cc} D:X\times G & \to \left[0,1\right], \end{array}$$

(9) $$\begin{array}{cc} A:G\times W & \to \left[0,1\right], \end{array}$$

are Markovian kernels (cf. Figure 2).

One can think of the experience space, (X,X), as pointing to an aware subject, which is aware whether or not it is having a specific experience. When there is no specific experience, then the aware subject enjoys awareness without content.

The set *X* points to the potential of this aware subject in having specific experiences. How this potential is actualized in specific experiences is not specified by the current theory of conscious agents. This is an important limitation to be addressed in future versions of the theory.

The σ-algebra X points to the conceptual representation of this aware subject about its own potential for having specific experiences. Since the σ-algebra X can be much smaller than the power set, P(X), the conceptual representation of the aware subject about its own potential can be simpler, even infinitely simpler, than its actual potential.

This conceptual representation of the aware subject need not be a self, but it may be among the building blocks of a self. The basic definition of a conscious agent does not include the notion of a self. We propose that a self must be constructed by networks of interacting conscious agents.

The set *G* points to the potential of the aware subject for acting. However, *G* is not necessarily experienced directly by the aware subject, because its experiences are limited to the set *X*.

For notational simplicity, we will not always explicitly mention the σ-algebras, writing, e.g., *X* to mean the pair (X,X) whenever obvious from context.

It is straightforward to show that networks of conscious agents are computationally universal. Anything that can be computed by neural networks or universal Turing machines can be computed by networks of conscious agents [67,68]. Thus, we expect to build networks of conscious agents that can construct a (simplified) model of themselves. Indeed, we expect to build networks for most items in the laundry list that started this section, including learning, memory, problem solving, intelligence, a self, free will, attention, combinations of qualia, combinations of subjects, morality, levels of awareness, altered states of consciousness, semantics, understanding, comprehension, the notion of a physical object, spacetime, quantum theory, and, finally, the relationship between physics and consciousness (including the hard problem of consciousness).

In the next Section 5, we look at one specific problem, namely the combination problem of consciousness. We have chosen this problem because it presents itself as arguably the most difficult problem for *any* theory in which consciousness is fundamental. We then move on to discussing the problem of how to conceive of physical objects within the theory of conscious agents (Section 6).

Those problems, we argue, are deeply related. A theory of combination (and “fusion” as we will introduce and discuss) defines, precisely, the basic ingredients we need to make sense of the notion of “physical objects in spacetime”. Our experience is immersive. We feel that we are inside spacetime and inside our bodies (which is also an object in spacetime). This feeling is not captured by the user interface metaphor. It is better captured if we switch to a virtual reality metaphor: we each wear a VR headset that presents us as an avatar immersed in a spacetime and interacting with objects. This allegory of the headset is just Plato’s allegory of the cave, with technology updated from fire to VR.

Before we turn to the combination problem, we briefly address a couple confusions that often arise about this program.

The interface theory of perception and the theory of conscious agents are distinct theories [69]. One might accept one and not the other (or reject both). However, if we accept both theories, they together entail that the distinction we make between conscious and unconscious objects is not principled, but is instead an artifact of the limitations of our perceptual interface. We say that a human being is conscious but a rock is not. However, if our perceptions of spacetime and objects are just an interface to a network of conscious agents beyond spacetime, then we are always interacting with conscious agents, no matter what object we see. In the case of a human being, when I look at a person’s face and body, I get some insight into their consciousness—are they happy, sad, fearful, relaxed, interested, distracted, and so on. When I look at a rock I get, say, hardness and brittleness, but minimal insights into consciousness. That doesn’t mean that I am not interacting with conscious agents. I always am. It’s just that my interface is giving me little insight into those agents. This is no surprise. The whole point of a user interface is to present a much simpler description, with much of the details of reality deleted, and to provide an adaptive representation of functional aspects of the interaction, so that one is not overwhelmed with too much information.

This entails, a fortiori, that the distinction we make between living and non-living objects is not principled. It too is merely an artifact of the limitations of our perceptual interface, and not an insight into the nature of reality. It should be no surprise, then, that attempts by scientists and philosophers to give a principled definition of life have so far failed. As the Stanford Encyclopedia of Philosophy says, in its entry on Life, “The literature on the definition of life is vast, repetitive, and utterly inconclusive. Philosophers have disagreed as to the ultimate source of the lack of consensus, citing unstated assumptions in either the definer’s approach or the question itself” [70].

The combination of the interface theory of perception with the theory of conscious agents has caused some to worry that they might be self-refuting. As the philosopher Philip Goff put it, “If we ought to doubt the testimony of our senses, then we ought similarly to doubt the testimony of our evolved capacity for forming judgements concerning the mental states of others. We are hardwired to judge the emotions of others on the basis of their behavior and facial expressions. However, if this hardwired capacity was evolved for survival rather than truth, and if this is sufficient for us to reject the deliverances of our sensory perception, then we ought likewise to reject the deliverances of our judgements about other minds. We ought to think others are zombies, or at least have no faith in our judgments that crying indicates sadness. I consider this a reductio ad absurdum of the ‘Fitness Beats Truth’ argument.” Similar reductio arguments are offered in [71].

The resolution of this apparent reductio ad absurdum comes from recognizing the two separate steps in our argument. The first step is a theorem about the theory of evolution by natural selection. That theory, for better or worse, entails that sensory systems are user interfaces, not windows on reality. However, that theory cannot answer the question, “Interfaces to what? What is the reality beyond our user interfaces?” To answer this question, we must take a new step, entirely separate from and beyond the theory of evolution by natural selection. We propose that the reality beyond our user interfaces is a reality composed of conscious agents.

On our proposal, our senses are interfaces to a network of conscious agents. This proposal goes beyond the confines of evolution by natural selection. In the definition of a conscious agent in Equation (6), we have introduced the σ-algebra X to stand for the conceptual representation of an aware subject. It is here where ITP demands caution. Almost certainly, X will not mirror any true structure of reality, such as when we sort what we experience into the categories of living/non-living. However, this does not mean that we do not undergo experiences *X*. In fact, we are having experiences every moment. Nor does it mean that experience is not being expressed in behavior. In fact, we do propose that *X* leads to *G* in our formalism. Hence, we should not expect others to be zombies or to not experience sadness when crying, as long as they (roughly) share our evolutionary history. It would probably be detrimental to our survival if we believed that others are zombies.

In short, it is a theorem that our perceptions of objects in spacetime is not a veridical perception of real objects in real spacetime; it is a user interface to a realm that is utterly unlike objects in spacetime. That realm happens to be a realm of conscious agents. It is no problem then to conclude that our interface gives real access to some of the conscious experiences of these agents.

## 5. Combine and Fuse

Can conscious subjects combine to form new conscious subjects? Can conscious experiences combine to form new conscious experiences? These are central questions of the *combination problem* of consciousness, famously raised in 1895 by William James in *The Principles of Psychology*:

“Where the elemental units are supposed to be feelings, the case is in no wise altered. Take a hundred of them, shuffle them and pack them as close together as you can (whatever that may mean); still each remains the same feeling it always was, shut in its own skin, windowless, ignorant of what the other feelings are and mean. There would be a hundred-and-first feeling there, if, when a group or series of such feelings were set up, a consciousness belonging to the group as such should emerge. Additionally, this 101st feeling would be a totally new fact; the 100 original feelings might, by a curious physical law, be a signal for its creation, when they came together; but they would have no substantial identity with it, nor it with them, and one could never deduce the one from the others, or (in any intelligible sense) say that they evolved it.” Further:

“Take a sentence of a dozen words, and take twelve men and tell to each one word. Then stand the men in a row or jam them in a bunch, and let each think of his word as intently as he will; nowhere will there be a consciousness of the whole sentence. We talk of the ’spirit of the age,’ and the ’sentiment of the people,’ and in various ways we hypostatize ’public opinion’. However, we know this to be symbolic speech, and never dream that the spirit, opinion, sentiment, etc., constitute a consciousness other than, and additional to, that of the several individuals whom the words ’age,’ ’people,’ or ’public’ denote. The private minds do not agglomerate into a higher compound mind”.

This combination problem has recently received substantial philosophical interest, propelled largely by the development of panpsychist theories of consciousness [20,72,73,74,75,76,77,78,79,80]. Seager describes the combination problem as “the problem of explaining how the myriad elements of ’atomic consciousness’ can be combined into a new, complex and rich consciousness such as that we possess” [72]. We use the theory of conscious agents to propose two mathematical approaches to the combination problem: *combination* and *fusion*.

To understand how conscious agents and qualia combine to create new agents and qualia, we study interactions among agents and how these interactions affect their experiences. To this end, we compose the kernels *P*, *D*, and *A* of an agent to create its “qualia kernel”, *Q* from *X* to itself (Using the definitions in Equations (7)–(9), the combined kernel Q=DAP is given, for x∈X and a measurable set B∈X, by $Q\left(x,B\right)=\int_{g\in G}D\left(x,dg\right)$$\left(\int_{w\in W}A\left(g,dw\right)P\left(w,B\right)\right)$):

(10) $$Q=DAP:X\times X\to \left[0,1\right].$$

*Q* describes the sequential experiencing of an agent, without reference to its other internal aspects, such as its decisions or actions, or to the nature of the network it is interacting with.

For kernels that have a matrix representation, composition is simply matrix multiplication. In this case, *Q* is a matrix that maps *X* to *X*.

The qualia kernel, *Q*, thus expresses the relation that conscious experience has to itself. This might also account for the elusive “what-it-is-likeness” of conscious experience. For the simplest agent, *X* has just one point x∈X, i.e., just one conscious experience. The qualia kernel would then give a Dirac measure on this point:

(11) $$Q\left(x,\cdot \right)=\delta_{x}\left(\cdot \right).$$

We call an agent with *n* distinct qualia an *n*-agent. Qualia can differ from one *n*-agent to another. We posit a large, perhaps countably infinite, set of 1-agents, each with a unique quale.

Now consider two 1-agents, $C_{1}$ and $C_{2}$, with qualia kernels

(12) $$Q_{1}:X_{1}\times X_{1}\to \left[0,1\right],$$

and

(13) $$Q_{2}:X_{2}\times X_{2}\to \left[0,1\right],$$

respectively. Let $X_{1}$ = $\left\{x_{1}\right\}$ and $X_{2}=\left\{x_{2}\right\}$, where $x_{i}$ denotes an experience.

**Figure 3:**
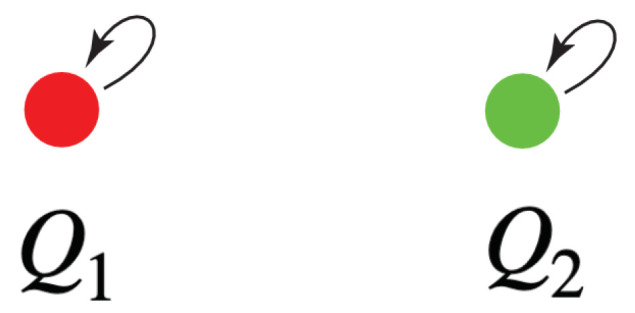
The qualia kernels of two 1-agents, one that experiences red and one that experiences green. In this diagram, the agents do not interact, and their qualia do not combine.

If we take $x_{1}$ to be the quale red and $x_{2}$ to be green, then we can depict the action of their qualia kernels as shown in Figure 3.

How might these agents interact and combine? To study this, consider the qualia kernel, *Q*, for the possible experiences of a 2-agent. This kernel is a 2×2 matrix whose entries are the following conditional probabilities:

(14) $$Q\left(x_{1},x_{2}\right)=\left[\begin{array}{cc} p(x_{1}|x_{1}) & p(x_{2}|x_{1})\\ p(x_{1}|x_{2}) & p(x_{2}|x_{2}) \end{array}\right],$$

We interpret the entry $p(x_{1}|x_{2})$ in this matrix as the probability that the next experience of the pair of agents will be red given that the current experience is green.

Suppose we have two agents

(15) $$C_{1}=\left(X_{1},G_{1},W_{1},P_{1},D_{1},A_{1}\right);\;C_{2}=\left(X_{2},G_{2},W_{2},P_{2},D_{2},A_{2}\right).$$

Observe that the 6-tuple

(16) $$C_{1}\times C_{2}=\left(X_{1}\times X_{2},G_{1}\times G_{2},W_{1}\times W_{2},P_{1}⊗P_{2},D_{1}⊗D_{2},A_{1}⊗A_{2}\right),$$

satisfies the definition of being a single conscious agent (cf. Equation (6) and [60]). We are assuming that the σ-algebras on the sets $X_{1}\times X_{2}$, etc., are the product algebras. The Markovian kernel $P_{1}⊗P_{2}:W_{1}\times W_{2}\to X_{1}\times X_{2}$ is defined by $P_{1}⊗P_{2}\left(w_{1},w_{2};dx_{1},dx_{2}\right)=P_{1}\left(w_{1};dx_{1}\right)P_{2}\left(w_{2};dx_{2}\right)$. $D_{1}⊗D_{2}:X_{1}\times X_{2}\to G_{1}\times G_{2}$ and $A_{1}⊗A_{2}:G_{1}\times G_{2}\to W_{1}\times W_{2}$ are defined similarly, and, thus, must be considered to be a single agent by the theory of conscious agents.

Hence, this juxtaposition of two simple agents leads to a more complex agent. The new agent then has two potential experiences, red and green. However, this new agent has a very simple *Q* kernel, namely $Q_{1}⊗Q_{2}$, which has a block-diagonal form. In particular, the off-diagonal terms, or cross terms, in Equation (14) are both zero, and we can think of agents $C_{1}$ and $C_{2}$ as “non-interacting”.

For now, we take it as an axiom that two agents can combine with arbitrary cross terms (as long as they fulfil the Markov criterion), as expressed in Equation (14). We conjecture that this axiom is not needed, and that for every legal set of cross terms, a network *W* can be constructed that would generate those cross terms.

If we let

(17) $$\begin{array}{cc} x & =p(x_{2}|x_{1}), \end{array}$$

(18) $$\begin{array}{cc} y & =p(x_{1}|x_{2}), \end{array}$$

then we can simplify (14) to

(19) $$Q\left(x,y\right)=\left[\begin{array}{cc} 1-x & x\\ y & 1-y \end{array}\right],$$

because each row needs to sum to 1 to satisfy the requirement of being Markovian.

If x=y=0, then this matrix is the identity matrix

(20) $$Q\left(x,y\right)=\left[\begin{array}{cc} 1 & 0\\ 0 & 1 \end{array}\right].$$

In this special case, where cross terms are 0, the two agents do not “interact.” Nevertheless, as we demonstrated in Equation (16), a pair of such non-interacting 1-bit agents, $C_{1}$, $C_{2}$, can be thought of as a single (combined) 2-bit agent. We will now show that two 1-agents cannot only be combined into a more complex 2-agent, but they can also “fuse” into a simpler 2-agent.

If either cross term is greater than 0, then the agents interact in a non-trivial dynamic. The maximum value of either cross term is 1. Thus, the set of kernels can be represented by points (x,y) in the unit square bounded by {(0,0),(0,1),(1,0),(1,1)}. This unit square, which contains all kernels with 2 states, we call the *Markov polytope*, $M_{2}$. The collection of all kernels with *n* states is a unit cube in *n*-dimensional real space and is here called the Markov polytope $M_{n}$.

The kernel *Q* describes a single interaction of the agents. To describe two consecutive interactions, we need the kernel $Q^{2}$, where

(21) $$Q^{2}=QQ=\left[\begin{array}{cc} {\left(1-x\right)}^{2}+xy & x(2-x-y)\\ y(2-x-y) & {\left(1-y\right)}^{2}+xy \end{array}\right]=\left[\begin{array}{cc} 1-x^{\prime } & x^{\prime }\\ y^{\prime } & 1-y^{\prime } \end{array}\right]$$

The difference quotient of these two kernels is the (in general non-Markovian) matrix $Q^{2}-Q$. We restrict attention to the off-diagonal terms of $Q^{2}-Q$, since they fix the diagonal terms. The off-diagonal terms define a discrete “kernel derivative,” dQ/dτ, with respect to a discrete-step parameter τ. This derivative, at a point (x,y) in the Markov polytope, $M_{2}$, is

(22) $$\frac{dQ}{d\tau }=\left(\frac{dx}{d\tau },\frac{dy}{d\tau }\right)=\left(x\left(1-x-y\right),y\left(1-x-y\right)\right).$$

**Figure 4:**
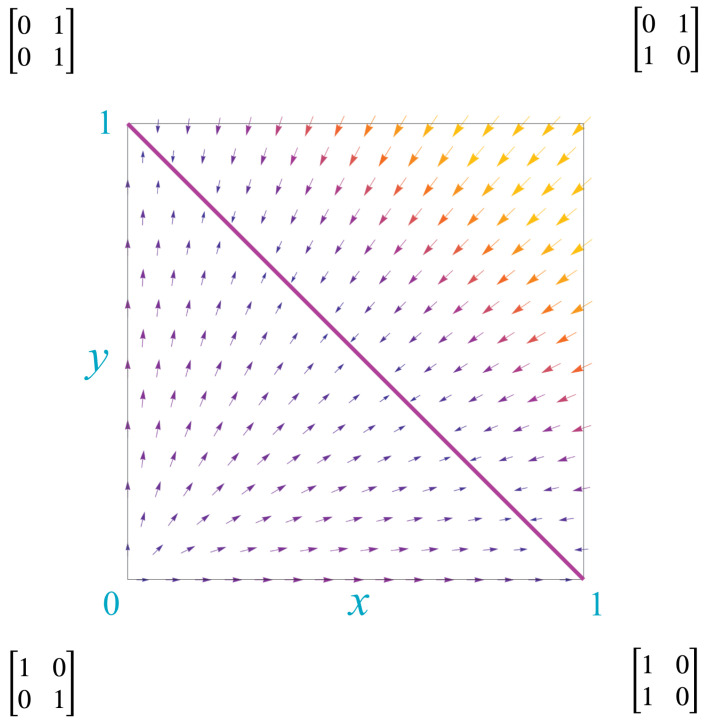
The flow of Markovian kernels on $M_{2}$, the Markov polytope of two states. This polytope is a unit square with coordinates (x,y), where $x=p(x_{2}|x_{1})$ and $y=p(x_{1}|x_{2})$. Arrowheads point in the direction of the local flow. Arrow length and color indicate the speed of flow.

The derivatives show the direction of “kernel flows” through $M_{2}$, as illustrated by the vector field in Figure 4. The “flow” of a Markovian kernel *Q* is the Markovian kernel $P=\lim_{n\to \infty }Q^{n}$.

The lower left corner of $M_{2}$, the point (0,0) corresponding to the identity matrix, is the single source for this vector field. The upper right corner, the point (1,1) corresponding to the NOT operator, does not flow but represents instead a periodic kernel with period 2. There is a line of sinks, the line y=1−x, depicted by the diagonal red line. Along this line, the kernels

(23) $$Q\left(x,y\right)=\left[\begin{array}{cc} 1-x & x\\ y & 1-y \end{array}\right]=\left[\begin{array}{cc} 1-x & x\\ 1-x & x \end{array}\right].$$

Thus, when kernels reach this line, they drop from rank 2 to rank 1. As we discuss later, this signals the fusion of agents and creation of new qualia.

Each kernel Q(x,y) has a unique combination of colors that it leaves invariant. We can represent a combination of colors by a probability measure

(24) $$\mu =\left[\begin{array}{cc} \alpha & 1-\alpha \end{array}\right],\;\;0\leq \alpha \leq 1,$$

where α is the proportion of red and 1−α is the proportion of green. The measure μ is invariant for the kernel *Q* if

(25) μQ=μ.

We can use this to find all kernels *Q* that have the same invariant measure.

(26) $$\left[\begin{array}{cc} \alpha & 1-\alpha \end{array}\right]\left[\begin{array}{cc} 1-x & x\\ y & 1-y \end{array}\right]=\left[\begin{array}{c} \alpha \\ 1-\alpha \end{array}\right].$$

Solving this we find the lines

(27) $$y=\frac{\alpha }{1-\alpha }x.$$

**Figure 5:**
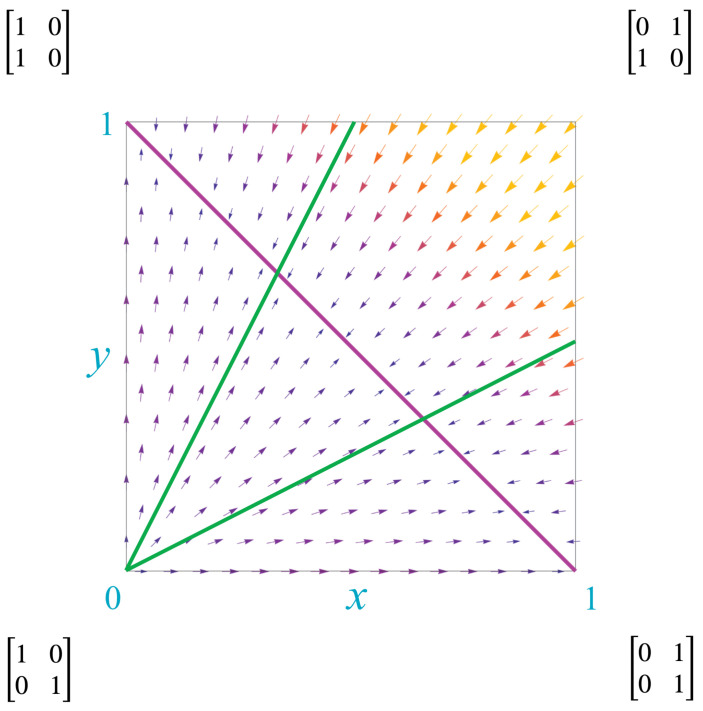
Invariant measures on the Markov polytope $M_{2}$. Kernels with the same invariant measure lie on a straight line passing through the origin. Two examples are shown as green lines. The lower line corresponds to $\alpha =\frac{1}{3}$. The upper line corresponds to $\alpha =\frac{2}{3}$.

Thus, for each value of α there is a line of kernels that follows the flow of the vector field. Two examples are shown in Figure 5, depicted as green lines.

**Figure 6:**
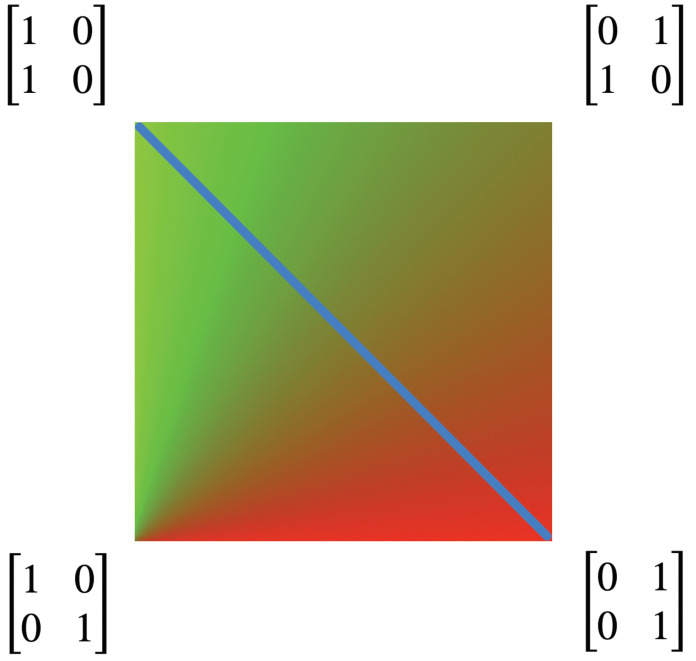
Invariant measures and stationary kernels in the Markov polytope $M_{2}$. The color at each point (x,y) indicates the mix of red and green in the invariant measure for the kernel Q(x,y) associated to that point by Equation (26). The blue diagonal line depicts the stationary kernels *Q*, satisfying QQ=Q, except for the identity matrix, which is trivially stationary.

It is helpful to visualize the invariant measure associated to each kernel in the Markov polytope $M_{2}$. This is completed in Figure 6, which depicts the mixture of red and green dictated by the invariant measure for each kernel in $M_{2}$. A stationary kernel is any kernel *Q* that is idempotent, i.e., that satisfies QQ=Q. These kernels correspond to the line of sinks shown in Figure 4. Figure 6 shows the relationship of these kernels to the mix of red and green in the invariant measures.

The stationary kernels lie on the line y=1−x. Along this line, the kernels can be written as:

(28) $$Q\left(x,y\right)=\left[\begin{array}{cc} 1-x & x\\ y & 1-y \end{array}\right]=\left[\begin{array}{cc} 1-x & x\\ 1-x & x \end{array}\right].$$

Thus, when kernels reach this line of stationary kernels, they drop from rank 2 to rank 1, and are a function of just one parameter, say

(29) α=x=1−y

**Figure 7:**
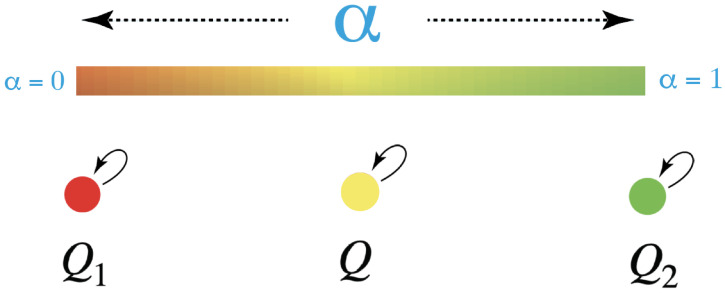
The unit 1-simplex of fusions for two conscious agents.

*This signals the fusion of agents*. The two agents, $Q_{1}$ and $Q_{2}$ first combine to form a two-parameter family, Q(x,y). All combined agents have the original two qualia: red and green. Now, with the drop in rank of the qualia kernel, the combined agents fuse to single agents with just one quale. There is a one-parameter family, indeed a unit 1-simplex, of new fused agents, Q(α), each with its own new quale, as illustrated in Figure 7.

**Figure 8:**
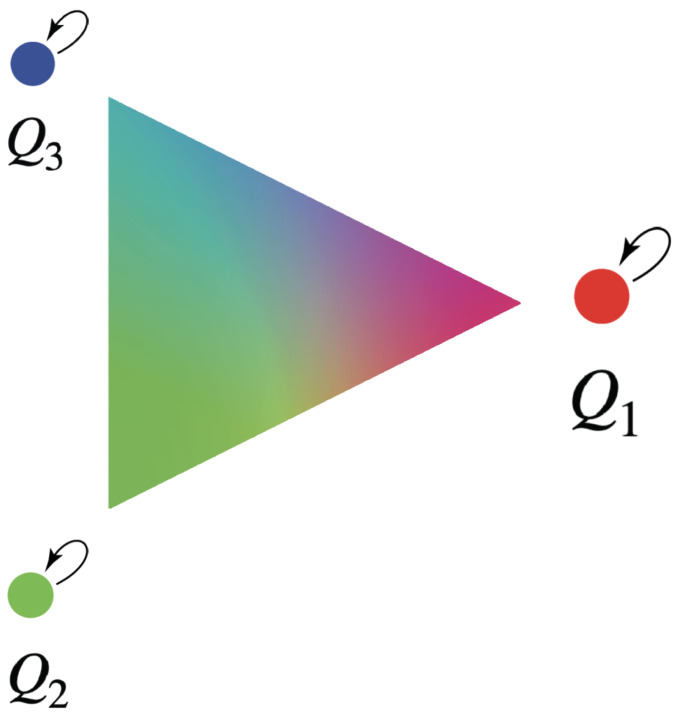
The unit 2-simplex of fusions for three conscious agents.

This pattern continues. If *n* conscious agents interact, their possible fusions form a unit (n−1)-simplex, the fusion simplex $F_{n}$, corresponding to the possible stationary kernels. For instance, if we have three agents, $Q_{1}$, $Q_{2}$, $Q_{3}$, with qualia red, green, and blue, respectively, then their possible fusions form a unit 2-simplex, illustrated in Figure 8.

Any collection of agents is itself an agent. Thus, there is ultimately one agent. A similar stance has been advocated by the physicist Erwin Schrödinger in his essay “Mind and Matter” [81]. Yet, the exploration of consciousness through its possible combinations and fusions appears to be endless. For this reason, a theory of consciousness cannot start with a theory of the “Ultimate One Consciousness.” However, if we start with a countable infinity, ${ℵ}_{0}$, of agents, then the number of possible combinations, $2^{n}$, and their fusions is a larger infinity, ${ℵ}_{1}$, and the number of possible new combinations of these combinations is yet a larger infinity, ${ℵ}_{2}$ and so on through Cantor’s hierarchy [82].

Viewed this way, the smaller units are perhaps nothing but projections of the One. If we could grasp the deep connection between these units and the One, we might at once resolve Schrödinger’s “arithmetic paradox” (the world is one, but the subject seems to be many, [81]) and also see the theory of conscious agents as a formal model of Leibniz’s monadology to document the “pre-established harmony” [65] between monads. However, for now, we must start with humble beginnings, and crawl up Cantor’s hierarchy. At each step in this process, our theory itself points to its inherent limitations, and to the infinite work undone. This antidote to dogmatism offers infinite job security for students of consciousness.

In the meantime, a next step is to study the possible combinations and fusions of three agents. We begin this study in the Appendix A to this paper.

## 6. Spacetime and Decorated Permutations

This section and the next propose how to project the dynamics of conscious agents down to spacetime, using structures called “decorated permutations.” We start by developing some necessary background.

How is consciousness related to spacetime and physical objects? Physicalist theories of consciousness usually assume spacetime and physical objects to be fundamental, and consciousness to be somehow emergent. By contrast, many panpsychist theories take consciousness to be the intrinsic nature of physical objects, and some seem to suggest that spacetime is fundamental. However, physics itself and evolution entail that spacetime is not fundamental, ruling out these theories.

The theory of conscious agents starts with a dynamics of agents that is, by hypothesis, outside of spacetime. So this theory must explain how spacetime and objects arise entirely from the dynamics of agents. This is a colossal project.

As Goff puts it, “It’s been hard enough to get the equations of physics we already have. Coming up with a whole new level of mathematical structure underneath that, which yields precisely the same predictions, is rather a large challenge. Moreover, I can’t see the motivation for taking on that challenge” [83].

“Physics” here is usually taken to refer to the physics of spacetime and objects, namely quantum field theory and general relativity. However, we do not locate the network of conscious agents *inside* spacetime precisely because physics and evolution tell us that these structures are not fundamental, and because physics itself has found new structures beyond spacetime, such as amplituhedra and their associated decorated permutations. So instead of merely re-describing conventional physics with conscious agents, we aim to show how decorated permutations, and other structures that physicists have found beyond spacetime, arise as a projection of a deeper theory of conscious agents.

An early hint of structures beyond spacetime came in 1986. Physicists study elementary particles by smashing them together at high energies and seeing what sprays out. Quantum field theory provides formulas, called scattering amplitudes, for computing the probabilities of various outcomes. These formulas derive from Feynman diagrams that model scattering as quantum processes occurring in spacetime, that is, as satisfying locality and unitarity. However, the formulas are complex. The formula for two gluons smashing to produce four gluons requires hundreds of pages of algebra. Then, in 1986, two mathematicians, Parke and Taylor, discovered a formula for gluon scattering that required only one term [84]. It did not model scattering as a process in spacetime, but pointed to a realm beyond spacetime.

The Parke–Taylor formula was soon followed by others that magically simplified the mathematics and pointed to a world beyond spacetime. Then in 2013, many of these results were unified into a single structure, a geometric object beyond spacetime, called the “amplituhedron” [22]. The amplitude of a particular scattering process is obtained by computing the volume of its corresponding amplituhedron. The amplituhedra for various scattering processes are faces of an infinite-dimensional master amplituhedron.

The amplituhedron is obtained via a linear mapping from a positive Grassmannian, [22,24,85]. Recall that the real Grassmannian G(k,n) is the space of all *k*-dimensional subspaces in an *n*-dimensional vector space. An element of G(k,n) can be represented, non-canonically, by any of its bases and therefore by a full-rank k×n matrix *C*. The Plücker coordinates for *C* are the determinants of all k×k minors of *C*. The positive Grassmannian, $G^{+}\left(n,k\right)$, is the subset of the real Grassmannian where all Plücker coordinates are non-negative, i.e., all subspaces have non-negative slope (see [85,86] for an informal discussion).

Remarkably, the invariant physical content of the positive Grassmanian is combinatorial, described by “decorated permutations”. Recall that a *permutation s* on the set $\bar{n}:=\left\{1,\ldots ,n\right\}$ is a bijection from the set to itself. We denote such a permutation by s=[s(1),s(2),…,s(n)].

An ordinary permutation *s*, say, can be *decorated* to yield mappings $\sigma :\bar{n}\to \bar{2n}=\left\{1,\ldots ,2n\right\}$ in the following ways: if s(a)>a, set σ(a)=s(a). If s(a)<a, set σ(a)=s(a)+n. If s(a)=a, set σ(a) to be either *a* or a+n. This suggests the

**Definition** **1.** *A decorated permutation is a mapping $\sigma :\bar{n}\to \bar{2n}$ such that, for any a,*

(30) a≤σ(a)≤a+n

*and*

(31) σ(a)mod(n)

*is an ordinary permutation.*

Notice that a decorated permutation thus defined is an injective mapping and is indeed a decoration of the (unique) ordinary permutation σ(a)mod(n). Moreover, we see that if an ordinary permutation has exactly *k* fixed points, there are $2^{k}$ decorated permutations corresponding to it. In particular, there are $2^{n}$ decorations of the identity.

**Figure 9:**
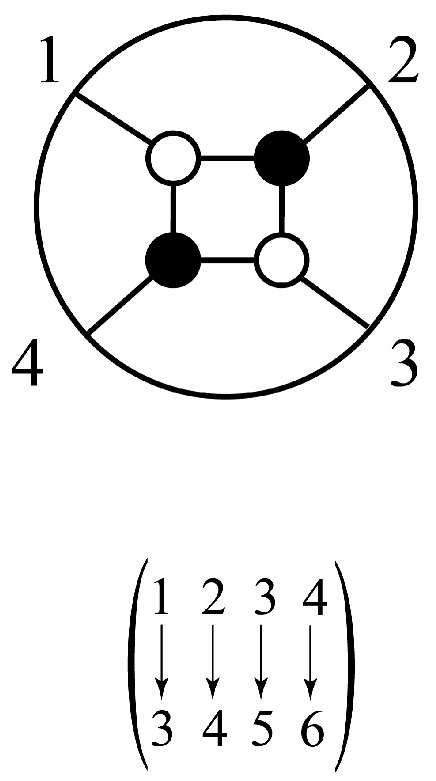
On-shell diagram for the decorated permutation σ=[3,4,5,6].

Decorated permutations can be represented by diagrams that mathematicians call “plabic graphs” and physicists call “on-shell diagrams” [22,87]. For instance, for the decorated permutation [3,4,5,6], an on-shell diagram is shown in Figure 9. The numbers to be permuted are arranged around a circle clockwise. To read off the permutation from the diagram, follow the line from a number into the diagram. If the line hits a white dot, turn left. If it hits a black dot, turn right. For instance, the line inward from 1 hits a white dot, so we turn left and hit a black dot, so we turn right and hit a white dot, so we turn left and arrive at 3. Thus, 1 is permuted to 3. Similarly, the line inward from 2 hits a black dot, so we turn right and hit a white dot, so we turn left and hit a black dot, so we turn right and arrive at 4. Thus, 2 is permuted to 4. If we start with 3, we will end up at 1. However, observe that, for the corresponding decorated permutation, we now have to add 4, because a=3>σ(a)=1, so we end up with 3→5. Analogously, we find 4→6.

In the other direction, for a given decorated permutation, there are many corresponding on-shell diagrams, of varying complexity. The diagram of relevance to physicists computing scattering amplitudes is a “reduced” diagram. It can be obtained by decomposing the decorated permutation into a minimal sequence of “adjacent” transpositions by the following simple algorithm, which can then be used to generate a corresponding on-shell diagram that is reduced [22]. For any decorated permutation σ that is not already a decoration of the identity permutation, find the lexicographically first pair of numbers in {1,…,n}, such that (1) a<c, (2) σ(a)<σ(c), and (3) the numbers between *a* and *c* are all mapped by σ to themselves, or to themselves plus *n*. Let (ac) denote the (ordinary) transposition of *a* and *c*: if it satisfies the three conditions above is called an *adjacent transposition*. Then we can decompose σ as $\sigma =\left(ac\right)\circ \sigma^{\prime }$. Repeat this process on the next available adjacent transposition. We can see that once all adjacent transpositions are exhausted, we will be left with the remaining permutation being a decoration of the identity. The final step from the product of adjacent transpositions to the minimal on-shell diagram is as follows: list the numbers 1,…,n above a rectangle. For the first transposition, say (ac), drop a line from *a* ending in a white circle and draw a horizontal line to a line dropped from *c*, ending in a black circle. Notice that, by our rules for the circles, one traces a path from *a* down to the white circle and turns left. Now there are two possibilities: (i) if *c* appears in a later transposition, extend the line downwards from the black circle (upon reaching the black circle one turns right): to a white circle if *c* is the lower number in the new transposition, a black circle if the higher number; (ii) if the state *c* is never again represented in one of the transpositions to follow, draw a line from the black circle back up to *c*. Continue until all transpositions are accounted for. Finally, if a number *b* is in no transposition, then σ(b)=b or σ(b)=b+n. In the first instance drop a line to a white circle and in the second to a black one. Finally, we simplify the diagram by eliminating bipartite circles.

**Figure 10:**
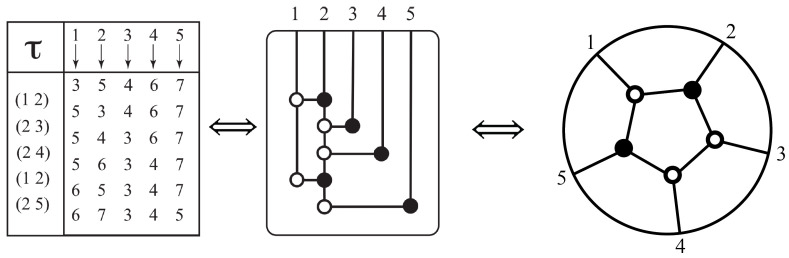
Construction of a reduced on-shell diagram in the middle corresponding to the decorated permutation [3,5,4,6,7]. On the right is a minimal version.

An example of this process is shown in Figure 10, where we see that the decorated permutation [3,5,4,6,7] is the decorated version of the ordinary product of adjacent transpositions: (12)(23)(24)(12)(25).

Note that we can recover all consistent decorated permutations from the diagram, by following these rules: starting at a state *i*, at any bipartite node, ignore it and continue straight through. At any tripartite node, turn left if the node is white and right if black. This path will end at some state. Then σ(i) equals this state if it is greater than *i*; if it is less than *i*, add *n* to obtain σ(i); and if σ(i)=i we can choose *i* or i+n.

This description of structures beyond spacetime is just an overview, and for brevity omits important structures, such as the associahedron and cosmological polytope [23,88]. If the theory of conscious agents is to explain how spacetime and objects arise entirely from the dynamics of agents, their combination and fusion, then it should somehow connect with these new structures beyond spacetime. This would allow the new physics to do some of the heavy lifting in building a bridge from consciousness to spacetime. Two key insights from physics guide our proposed connection.

One key insight is this: the deepest structure beyond spacetime that distills physics is the decorated permutation. From decorated permutations, one can construct reduced on-shell diagrams. Differential forms on these on-shell diagrams give rise to the scattering amplitudes. (Without supersymmetry one also needs helicities, or masses and spins [24]).

A second key insight is that any on-shell diagram arises from combining diagrams containing single three-legged black or white dots. They are the only diagrams for three-particle interactions, which are sufficient for computing *all* interactions [22].

These two insights give a clear target for the theory of conscious agents: its Markov-chain dynamics must map to decorated permutations and spins. With that map, we can propose a precise correspondence between (1) the Markov polytopes that describe all possible agent dynamics and (2) the on-shell diagrams that generate scattering amplitudes.

So, what is the map from Markov chains to decorated permutations? There is recent work relating Markov chains, positive Grassmannians, and amplituhedra [85]. However, the map from Markov chains to decorated permutations has been an open problem.

## 7. Correspondence between Agent Dynamics and Physical Particles

In a Markov chain with kernel *Q*, we say that state *a communicates with* state *b* if there is a positive probability that the chain starting at *a* reaches *b* in finite time: we have $Q^{j}\left(a,b\right)>0$ for some natural number j≥0. Given a state $a\in \bar{n}:=\left\{1,\ldots ,n\right\}$, its *communicating class* $\left[a\right]$ consists of all states *b* such that *a* and *b* communicate with each other. Mutual communication is an equivalence relation, so the communicating classes of a given kernel partition the state space. Since the state *a* communicates with itself (set j=0 above), the singleton {a} is always a subset of the communicating class [a]. When [a]={a}, i.e., when Q(a,a)=1, we say that *a* is *absorbing*: the chain, starting at *a*, stays at *a*. More generally, if the chain returns to *a* infinitely often with positive probability, we say that *a* is *recurrent*. If a state is not recurrent, it is *transient*: the probability of its returning to *a* infinitely often is zero, and this is equivalent to having $\sum_{j=0}^{\infty }Q^{j}\left(a,a\right)<\infty$ (cf. Theorem 1.5.3 of [89]).

**Definition** **2.** ***Markov Decorated Permutations.*** *Given a Markov kernel on $\bar{n}$, we define the decorated permutation $\sigma :\bar{n}\to \bar{2n}$ as follows:*

- *If a is transient, set σ(a)=a.*
- *If a is recurrent, let σ(a) be the first element b>a of {1,…,2n}, such that the sequence (a,a+1…,b) between a and b contains the communicating class $\left[a\right]$ (recall that a number c>n represents the state cmod(n)).*

*In both instances, a≤σ(a)≤a+n; moreover σ(a) mod(n) is bijective, so we indeed have a decorated permutation. Notice, also, that when a is absorbing, we have σ(a)=a+n.*

For instance, a Markov chain on 9 states in which the cycles (including cycles of period 1) are (158), (2), (34), (6), and (79) would be represented by the decorated permutation [8,11,4,12,10,15,9,14,16]. If state 2 were transient, then the permutation would be [8,2,4,12,10,15,9,14,16]. We note, in passing, that the assignment of decorated permutations to Markov chains is easily generalized to an assignment of decorated permutations to arbitrary graphs, a generalization that may prove useful for the analysis of networks.

**Definition** **3.** ***Decorated Permutations of Arbitrary Graphs***. *Given an arbitrary graph whose set of nodes is $\bar{n}$, define the decorated permutation for each $a\in \bar{n}$ as follows: (1) if a has no links, then set σ(a)=a; (2) if a only links with itself, then set σ(a)=a+n; and (3) otherwise, let σ(a) be the first element b>a of (1,…,2n), such that (a,…,b) contains all nodes in the strongly connected component of the graph containing a. (Recall that a number c>n represents the node cmod(n), and that a strongly connected graph has a path from each node to every other node).*

**Figure 11:**
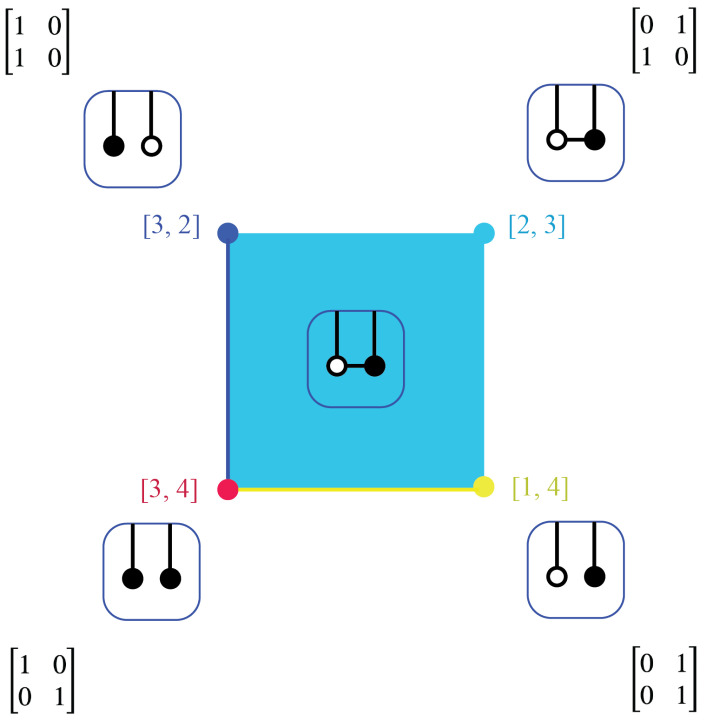
The cell complex for $M_{2}$ (colored disks, edges, and interior of the square) and the associated on-shell diagrams (black and white dots).

The Markov polytope $M_{n}$ is a cell complex. The cell $S_{\sigma }$ is the set of all Markov kernels $K\in M_{n}$ which give rise to the decorated permutation σ. For instance, $M_{2}$ has four cells, as shown in Figure 11. The identity kernel, depicted by a red disk, is a cell by itself, with σ=[3,4], a decoration of the identity indicating that each state maps to itself. The NOT-kernel, depicted by a light blue disk, and the entire interior of $M_{2}$ is a cell, with σ=[2,3], indicating a communicating class of size 2. The segment y=0 minus the origin, depicted in yellow, is a cell, with σ=[3,2]. The segment x=0 minus the origin, depicted in dark blue, is a cell, with σ=[1,4]. Figure 11 also shows on-shell diagrams associated to each cell in $M_{2}$.

Decorated permutations code for subspaces of the positive Grassmannian as follows. For a k≤n, let *C* be a k×n matrix of full rank *k*, representing an element of the positive Grassmannian $G^{\geq 0}\left(k,n\right)$ (Recall that the Grassmannian G(k,n) is the set of *k*-dimensional subspaces of $R^{n}$. Any such subspace may be represented by such a matrix *C*, whose *k* rows are a basis of that subspace: at least one k×k minor is non-zero. The *positive Grassmannian* $G^{\geq 0}\left(k,n\right)$ consists of those subspaces that can be represented by a matrix *C all* of whose k×k minors are non-zero and of the same sign. All representative matrices of such a subspace will then share this property). Repeat the *n* columns of *C*, in order, to produce a k×2n matrix $C^{*}$. The decorated permutation $\sigma_{C}$ associated to *C* assigns to each column number a∈{1,…,n} the value $\sigma_{C}\left(a\right)>a$ as follows: if $c_{a}\neq \vec{0}$, $\sigma_{C}\left(a\right)$ is the first column *b* of the expanded matrix $C^{*}$, such that $c_{a}$ is in the span of $\left\{c_{a+1},\ldots ,c_{b}\right\}$. If, however, $c_{a}=\vec{0}$, define $c_{\sigma }\left(a\right)=a$.

Clearly, the set of full rank k×n matrices with a given decorated permutation corresponds to a unique subspace of $R^{n}$ and vice versa. Thus, a decorated permutation that codes for a communicating class of size *l* of a Markov chain also codes for a subspace of dimension l−1 in the positive Grassmannian. Since communicating classes dictate the asymptotic behavior of Markov chains, this correspondence between communicating classes and subspaces may underlie the unexpected finding that the stationary distribution of the asymmetric simple exclusion process depends on the combinatorics of cells of the positive Grassmannian [90], and that regular soliton solutions of the KP equation, governing interaction patterns of shallow waves, depend on the cell of the positive Grassmannian that the starting point lies in [91]. In turn, the positive Grassmanian maps into an amplituhedron, on whose faces the computations of physical interactions are then made.

This has guided our search for a correspondence between the dynamics of conscious agents and the dynamics of scattering in spacetime.

Recalling Figure 4, notice that, with the exception of the NOT corner, the cells of Figure 11 are precisely the segmentation of $M_{n}$ into its different possible flow patterns. This suggests that we think of the map from $M_{n}$ to its cells, which are communicating classes, as a projection via its dynamics to their associated decorated permutations. Physics, as we have noted above, finds fundamental significance in decorated permutations: they lead from their on-shell diagrams to faces of the amplituhedron and the differential forms on those faces that yield the scattering amplitudes. These last are a complete description of *any* physical energetic exchange at the micro-level. Physical scattering events are then explained in terms of “particles” in spacetime, so these ideas suggest the conjecture that this procession, or “projection,” from at least some of the cells of $M_{n}$, have meaning as particle interactions in physics: if so, let us call such projections *physical*. That is, we conjecture an *agent–particle correspondence*: a particle (in spacetime) is an aspect of a physical projection of the dynamics of a communicating class of conscious agents to a face of an amplituhedron.

The smallest non-trivial communicating class is a single conscious agent. So the Markov polytope $M_{j}$, describing all possible dynamics of the conscious *j*-agent, is the smallest Markov polytope that may have projections onto the dynamics of *j*-particle scattering in spacetime. All Markov polytopes $M_{k}$, with k>j, may also have projections onto the dynamics of *j*-particle scattering.

**Figure 12:**
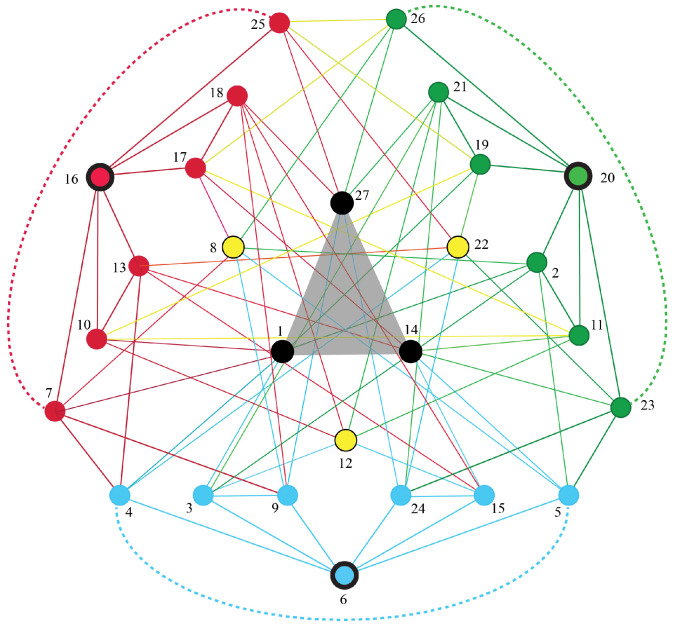
The adjacency graph for the Markov polytope $M_{3}$. Vertices are numbered to correspond with Appendix B. The grey triangle represents the total fusion simplex. Blue dots are vertices of the partial fusion complex.

As a specific example of this agent-particle correspondence, consider the Markov polytope $M_{3}$, which describes all dynamics of conscious 3-agents. Appendix B presents the 17 distinct decorated permutations for the 27 vertices of $M_{3}$. The adjacency graph for $M_{3}$ is shown in Figure 12. Appendix A presents some of the geometric structure of $M_{3}$.

**Figure 13:**
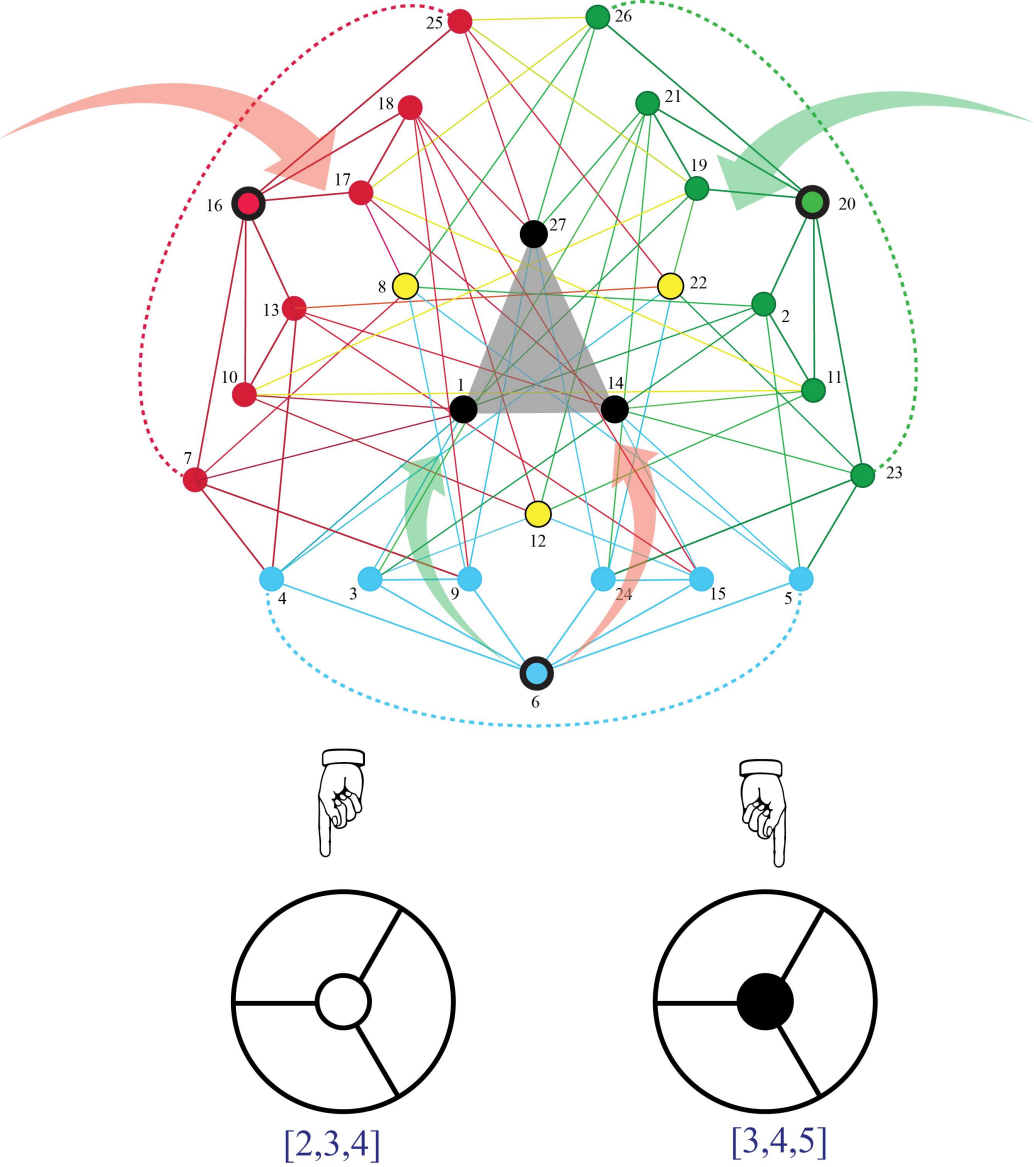
Flows in the Markov polytope $M_{3}$. Computer simulations indicate that, analogous to the situation in $M_{2}$, there are two types of flow on either side of the total fusion simplex coming from the identity (6) on one hand and the two maximal derangements (16 and 20) on the other. We conjecture that these two types of flows are in the relation indicated (by the pointing hands) to the pair of 3-particle amplitudes of scattering theory.

In this case, $M_{3}$ is the smallest Markov polytope with projections onto the two possible on-shell diagrams for three-particle interactions shown in Figure 13. We conjecture that the fusion simplex thereby defines two types of flows that correspond to the pair of 3-particle amplitudes of scattering theory. It will be intriguing to see how physical properties such as mass, momentum, and spin arise as projections from Markov polytopes.

## 8. Discussion

Newtonian physics is a beautiful theory, with practical applications that are explored and exploited to this day. However, it is not fundamental. In 1905, with the publication of Einstein’s paper on special relativity, its reign ended. For those seeking a fundamental theory, it was time to move on.

However, scientific theories have their own momentum. In 1922, when Einstein received his Nobel Prize, the committee noted that the award was given “without taking into account the value that will be accorded your relativity and gravitation theories after these are confirmed in the future” [92].

Quantum field theory and Einstein’s theory of gravity are beautiful theories, with practical applications that are likely to be explored and exploited for centuries to come. However, they, and spacetime itself, are not fundamental. For those seeking a fundamental theory, it is time to move on. Physics has indeed moved on, proposing, e.g., string theory [93], loop quantum gravity [94], causal sets [95], amplituhedra [22], and cosmological polytopes [23,88].

However, scientific theories of consciousness, and the combination problem, have not moved on. Physicalist, dualist, and panpsychist theories assume that spacetime is fundamental. Functionalist theories could, in principle, eschew spacetime and seek instantiation elsewhere [96]. However, functionalists tacitly, and often explicitly, assume an instantiation within spacetime. We propose that, as long as functionalists seek instantiation within spacetime, the question why and how it feels for something to have a mind will forever elude them.

Spacetime recidivism has consequences. No theory in the scientific study of consciousness, to date, accounts for any specific conscious experience. What integrated information must be the taste of chocolate and could not be the taste of vanilla? What orchestrated collapse of quantum states must be the smell of coffee and could not be the smell of coconut? What state of a global workspace must be the sound of a harp and could not be the sound of a flute? No answer has yet been given. This failure was foreseen by Leibniz in his famous Mill Argument: “It must be confessed, however, that Perception, and that which depends upon it, are inexplicable by mechanical causes, that is to say, by figures and motions. Supposing that there was a machine whose structure produced thought, sensation, and perception, we could conceive of it as increased in size with the same proportions until one was able to enter into its interior, as he would into a mill. Now, on going into it he would find only pieces working upon one another, but never would he find anything to explain Perception. It is accordingly in the simple substance, and not in the composite nor in a machine that the Perception is to be sought. Furthermore, there is nothing besides perceptions and their changes to be found in the simple substance. Additionally, it is in these alone that all the internal activities of the simple substance can consist” [65]. We agree with Leibniz. Any theory that reduces consciousness to mechanisms involving states, configurations, or processes of objects will fail to account for any specific conscious experience.

Steven Pinker acknowledges this failure: “Our best science tells us that consciousness consists of a global workspace representing our current goals, memories, and surroundings, implemented in synchronized neural firing in fronto-parietal circuitry. However, the last dollop in the theory—that it subjectively feels like something to be such circuitry—may have to be stipulated as a fact about reality where explanation stops” [97]. Indeed, current theories of consciousness do not explain conscious experience, they stipulate it.

Pinker explains why. “This should not be entirely surprising. As Ambrose Bierce noted in The Devil’s Dictionary, the mind has nothing but itself to know itself with, and it may never feel satisfied that it understands the deepest aspect of its own existence, its intrinsic subjectivity” [97]. Indeed, it may be that subjective experience cannot be explained, and must be stipulated. If so, then let us stipulate it and, with a nod to William of Ockham, nothing else. Stipulate a dynamics of experiences and derive, rather than stipulate spacetime and objects as a projection of the dynamics. This is the project of the theory of conscious agents. To that end, this paper proposes a map from agent dynamics through the amplituhedron into spacetime.

This approach agrees with Pinker’s next point. “Whatever we make of the hard problem of consciousness, positing an immaterial soul is of no help at all. For one thing, it tries to solve a mystery with an even bigger mystery. For another, it falsely predicts the existence of paranormal phenomena” [97]. Indeed, positing an immaterial soul offers no formal theory, and thus no help to a science of consciousness. It introduces a dualism, and thus entails paranormal phenomena. The theory of conscious agents, by contrast, is monistic and precise. Consciousness is no ghost in the machine. Instead, the laws of physics and the special sciences are themselves a projection of the dynamics of conscious agents. New laws may emerge from the study of conscious agents. However, these will be formal advances with testable consequences—such as the move from classical to quantum theorie—not ad hoc posits.

In this paper, we sketched how spacetime and particles may arise as a projection of the dynamics of conscious agents. By itself, this does not tell us anything about the *experience* of time, which flows from a definite past to a present moment towards an open future. It is instructive therefore to see how an entropic, i.e., a forward-directed, time might similarly arise in the theory of conscious agents.

It is straightforward to construct a homogeneous Markovian dynamics, *X*, of conscious agents with constant entropy, $H(X_{n})$. That is,

(32) $$H\left(X_{n}\right)=H\left(X_{n-1}\right),\;\;\;\forall n.$$

Here, *n* refers to the number of updating steps in the dynamics of conscious agents (cf. Equation (6)), and not to the number of agents involved.

This dynamics has no preferred direction. However, any projection of this dynamics via conditional probability induces an entropic arrow of time [98]. That is, if we condition, say, on $X_{1}$, then

(33) $$H\left(X_{n}|X_{1}\right)\geq H\left(X_{n-1}|X_{1}\right),\;\;\;\forall n.$$

**Proof.**  The proof is straightforward. We note that

(34) $$H\left(X_{n}|X_{1}\right)\geq H\left(X_{n}|X_{1},X_{2}\right).$$

(Conditioning reduces uncertainty). However, by the Markov property we have

(35) $$H\left(X_{n}|X_{1},X_{2}\right)=H\left(X_{n}|X_{2}\right),$$

and by homogeneity,

(36) $$H\left(X_{n}|X_{2}\right)=H\left(X_{n-1}|X_{1}\right).$$

The last three equations imply that Equation (33) holds. □

Thus, the theory of conscious agents may find that entropic time is not a fundamental feature of reality, but merely an artifact of projection. To explain the “arrow of time” as an emergent property of entropic systems is not a new idea, e.g., [99,100]. However, in contrast to most approaches, we do not assume that our experience of an arrow of time somehow mirrors physical evolution (in fact, evolution by natural selection tells us this is most unlikely to happen), but that it arises as a projection of a dynamics of consciousness that *underlies* physical evolution. The same formalism (i.e., Markovian networks of conscious agents) that could explain scattering events in spacetime could also shed light on experienced temporality.

In evolution, organisms compete for resources to counter the ravages of entropy. The fundamental limited resource is time. Could it be that this entire evolutionary framework is an artifact of projection from the dynamics of conscious agents in which there are no limited resources and no competition? This becomes a technical question in the theory of conscious agents, requiring a specification of the dynamics of agents and the precise mapping of this dynamics into spacetime. We would require to obtain evolution as a projection of agent dynamics with the same rigor in which we get, say, classical physics as a limiting case of quantum.

## Figures and Tables

**Figure A1:**
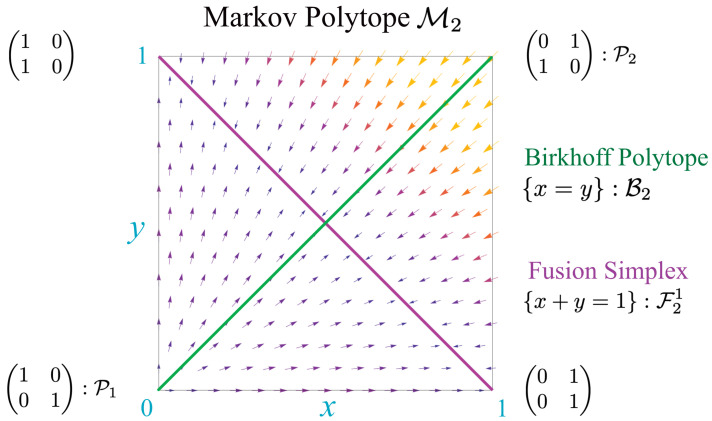
Parameter space for $M_{2}$ with fusion polytope, Birkhoff polytope, periodic points, and dynamics.

**Figure A2:**
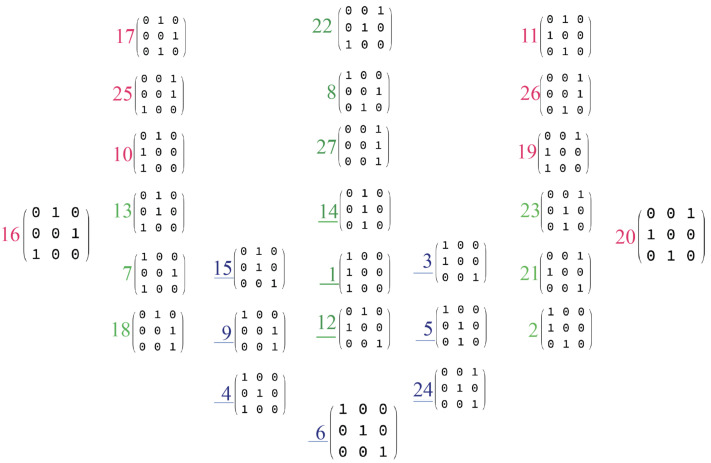
Vertices of $M_{3}$.

**Figure A3:**
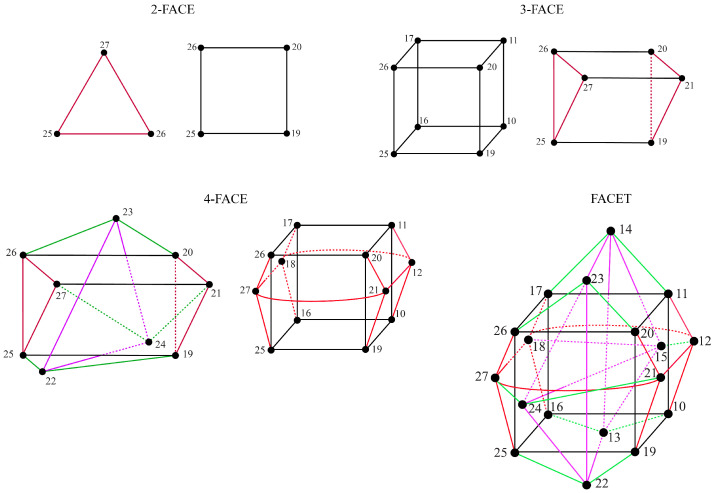
Examples of the types of *r*-faces of $M_{3}$ of dimension r=1,…,5. Numbering as in Figure A2. There are two 4-Faces, with 6 and 8 vertices, respectively: their own faces can be seen to include the prior 3-faces, additionally surrounded by and connected to, respectively, an outer “enveloping” triangle; or two prisms connected by a square. The 5-facet is generated by the 12-vertex 4-face with two purple enveloping triangles (13-14-15 and 22-23-24) and their connections to the 4-face and each other.

**Figure A4:**
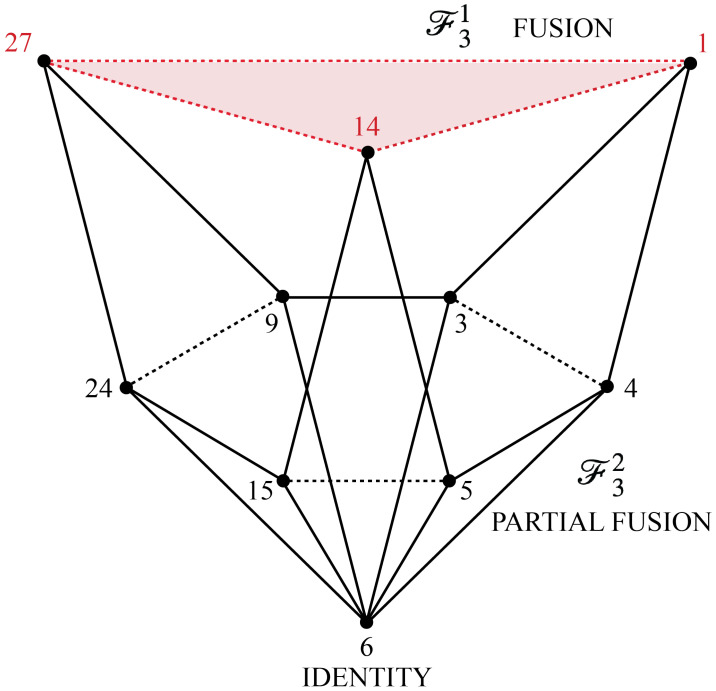
The stationary stochastic elements of $M_{3}$. Solid lines are edges; dotted lines are segments internal to a square face of $M_{3}$. The fusion polytope $F_{3}^{1}$ is a 2-dimensional simplex while $F_{3}^{2}$, the partial fusion complex, is the union of 6 segments.

**Figure A5:**
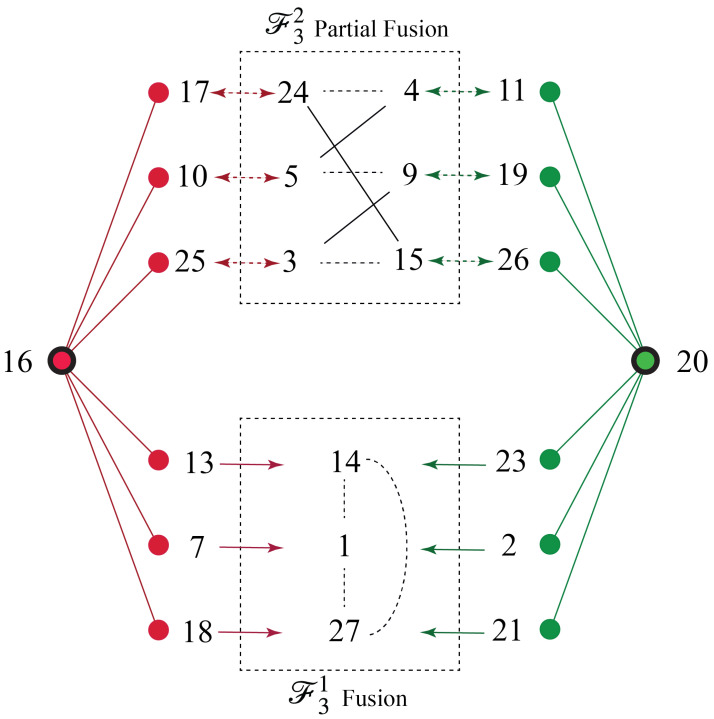
Caps and their dynamics in $M_{3}$. Solid lines and arrows are edges of $M_{3}$, dashed ones are not. Single solid arrows indicate that powers of the tail matrix becomes the head matrix. For example, $13^{n}=14,n\geq 2$. Double-dashed arrows indicate that powers of the cap matrices of 16 and 20 alternate between itself and the partial fusion matrix at the other end. For example, $11^{2n+1}=11$ and $11^{2n}=4.$

## Abbreviations

The following abbreviations are used in this manuscript:

ITP	The Interface Theory of Perception

## Appendix A. Geometry and Dynamics of Markov Polytopes

In order to understand agent fusion and its possible connections with particle physics, we need to study the geometry and dynamics of Markov polytopes $M_{n}$, of arbitrary order *n*. In this Appendix, we make a start towards such a study. We give some definitions and facts for general *n*, but mostly focus, as in the body of this paper, on the instances $M_{2}$ and $M_{3}$, respectively, of two and three interacting conscious agents. Proofs of some assertions are provided in the last subsection.

Our main goal here is to demonstrate how enormously richer, already, is the 3-agent theory than that for 2-agents; the analysis here is inspired by the considerations in the body of this article. Here, we take a naïve approach to the analysis; we anticipate in future work a deeper study, for arbitrary *n*, applying the general theory of convex polytopes [101,102,103]. In addition to delineating aspects of the geometry of $M_{3}$, we have made a start in identifying the connection between the geometry and asymptotic dynamics. We hope to announce later further results on this, both by use of computer simulation and by theoretical analysis.

### Appendix A.1. Geometric Structure of Markov Polytopes

An n×n matrix is *stochastic*, a *Markov kernel* or a *transition matrix*, if its entries are real numbers between 0 and 1 and its rows sum to 1. We can think of a stochastic matrix as the transition matrix of a Markov chain, on the *state space* $\bar{n}:=\left\{1,\ldots ,n\right\}$. For a given *n*, the set $M_{n}$ of all such matrices forms a compact, convex set in $R^{n^{2}}$ under the usual operations of matrix addition and scalar multiplication. The *extreme points*, or *vertices*, of a convex set are those of its elements which are not convex combinations of any others. In $M_{n}$ the vertices are those stochastic matrices each of whose rows is a *unit* row: a row with a single 1 entry (and the remaining entries all 0). It can be seen that $M_{n}$ is the set of the set of convex combinations of its vertices. Hence, we call $M_{n}$ the *Markov polytope of degree n* (As the vertices have all integral coordinates, it is an *integral* polytope, and we will see that it is also *simple*: each of its vertices is connected by boundary edges to a number of other vertices equal to the dimension n(n−1) of $M_{n}$).

For instance, elements of $M_{2}$ and $M_{3}$ can be described, respectively, by matrices of the form

(A1) $$\left[\begin{array}{cc} 1-a & a\\ b & 1-b \end{array}\right],\;\left[\begin{array}{ccc} 1-a-b & a & b\\ d & 1-c-d & c\\ e & f & 1-e-f \end{array}\right],$$

where all entries are between 0 and 1. For a 2×2 stochastic matrix there are two parameters to be specified; $M_{2}$ is thus the image, under a 1:1 affine map, of a 2-dimensional domain manifold: the Cartesian product of 2 copies, one for each row, of the standard simplex $S_{1}$ of dimension 1. $M_{2}$ is thus a 2-dimensional manifold in $R^{2\times 2}≃R^{4}$. Similarly, $M_{3}$ is the image, under a 1:1 affine map, of a 6-dimensional domain: the Cartesian product of 3 copies, one for each row, of the standard simplex $S_{2}$ of dimension 2. Thus, $M_{3}$ is a 6-dimensional manifold in $R^{3\times 3}≃R^{9}$. For convenience, Figure A1 reproduces the depiction of $M_{2}$ given earlier in Figure 4.

Consider the general polytope $M_{n}$. For i,j denoting integers in $\bar{n}$ we define the *parameter space* $\Pi_{n}:=\left\{{\left(x_{j}^{i}\right)}_{i\neq j}|\;x_{j}^{i}\geq 0;\forall i,\sum_{j=1,j\neq i}^{n}x_{j}^{i}\leq 1\right\}$, which is a subset of ${ℜ}^{n(n-1)}$. A bijective mapping $\Pi_{n}\to M_{n}$ is then given by

$${\left(x_{j}^{i}\right)}_{i,j=1,\;i\neq j}^{n}\mapsto \left[\begin{array}{ccccccc} \left(1-\sum_{j\neq 1}x_{j}^{1}\right) & x_{2}^{1} & \ldots & \ldots & \ldots & x_{n-1}^{1} & x_{n}^{1}\\ \ldots & \ldots & \ldots & \ldots & \ldots & \ldots & \ldots \\ x_{1}^{i} & x_{2}^{i} & \ldots & \left(1-\sum_{j\neq i}x_{j}^{i}\right) & \ldots & x_{n-1}^{i} & x_{n}^{i}\\ \ldots & \ldots & \ldots & \ldots & \ldots & \ldots & \ldots \\ x_{1}^{n} & x_{2}^{n} & \ldots & \ldots & \ldots & x_{n-1}^{n} & \left(1-\sum_{j\neq n}x_{j}^{n}\right) \end{array}\right]$$

In general, $M_{n}$ is thus an affinely embedded manifold of dimension n(n−1) in $R^{n\times n}≃R^{n^{2}}$. Again, it is the convex hull of its extreme points, or vertices. These are the matrices with all unit rows, i.e., rows with a single unit entry: thus, there are $n^{n}$ vertices in $M_{n}$. The 4 vertices of $M_{2}$ are shown in Figure A1. The 27 vertices of $M_{3}$ are arrayed in Figure A2 (The numbering was assigned by a program in ™MATHEMATICA to systematically generate them starting from No. 1; we have arrayed and colored the matrix numbers by “distance” from the identity, i.e., the number of rows in the identity that need to be changed to reach the new matrix: blue means one row, green means two, red means three rows. This numbering turns out to be somewhat prescient: see Proposition 2 and the example before it).

Given a set *X* of points in a vector space, we will say that its convex hull C(X) is *generated* by that set. As $M_{n}$ is a convex polytope, its boundary, denoted $\partial M_{n}$, consists of *faces*: maximal flat regions generated by a subset of vertices. These are polytopes in their own right and their faces are subsets of those of the mother polytope. Amongst the faces, those of 0 dimension are the vertices, or 0-*faces*, 1-dimensional faces are *edges*, or 1-*faces*, those of 1 dimension less than the maximum are the *ridges*, while those of maximal dimension are termed *facets*. The *dimension* of a polytope is 1 more than the dimension of its facets (This notion of dimension is equivalent to the usual definition of dimension: a convex polytope is of dimension *r* if it is homeomorphic to the *r*-dimensional unit ball).

Two vertices are *adjacent* if they are connected by an edge of the polytope: the line segment generated by them is not contained in the interior of any other face; equivalently, that none of its points are convex combinations of any other pair of vertices. Each face is also a polytope; its own faces are amongst the faces of $M_{n}$. If two faces share a common (lower-dimensional) boundary face, we say that they *abut* one another.

**Proposition** **A1.**
*Two vertices are adjacent if they differ only in the position of a unit entry in the same row, the other rows being identical.*

The set of vertices adjacent to a given vertex *V* will be termed the *cap* $C_{V}$ of *V*. As in Figure A1 the caps in $M_{2}$ have 2 vertices each and we will see that the caps of $M_{3}$ have 6 vertices each. By Proposition A1, the caps of $M_{n}$ have n(n−1) vertices each.

The description above of a polytope as the convex hull of its vertices is termed the V-description. An equivalent description is the H-description, in terms of the set of half-spaces bounding the polytope. Each such half-space is a linear inequality in the coordinates of the parameter space (for $M_{n}$, this is $R^{n(n-1)}$) and the faces of the polytope are given by specifying certain subsets of these as *equalities*, with the proviso that the resulting set is bounded. For example, recalling Equation (A1), $M_{2}$ can be described as the set bounded by four half-spaces, which we can separate into two groups of two inequalities each:

a≥0,1−a≥0

and

b≥0,1−b≥0

We get a face of $M_{2}$ by converting, into equalities, *one* of the inequalities in at least one group, while keeping the remaining inequalities as is. If this is performed in both groups, we get one of four different 0-faces; if in exactly one group, we get one of four different 1-faces, the facets of the 2-dimensional polytope $M_{2}$. This is depicted in Figure A1.

To find the faces of $M_{3}$, we follow the same principle on its three bounding groups of inequalities, by converting into equalities, within at least one group, no more than *two* of its inequalities (since if n−1 entries in a row of a stochastic n×n matrix are zero, the remaining entry has to be 1). Recalling the second matrix of Equation (A1), the inequality groupings are

a≥0,b≥0,1−a−b≥0

(A2) c≥0,d≥0,1−c−d≥0

e≥0,f≥0,1−e−f≥0

So now we can distinguish the following possibilities for the different types of faces of each dimension in $M_{3}$. Figure A3 depicts examples of each type. The types are exemplified in Example A1 and Proposition A2 gives details of the shapes and numbers of each type.

**Example** **A1.**
**Faces of $M_{3}$**

- *C{25} is of the form $\left[\begin{array}{ccc} 0 & 0 & 1\\ 0 & 0 & 1\\ 1 & 0 & 0 \end{array}\right]$, which generates a 0-face or vertex*
- *C{25,27} is of the form $\left[\begin{array}{ccc} 0 & 0 & 1\\ 0 & 0 & 1\\ e & 0 & 1-e \end{array}\right]$, which generates a 1-face, or edge*
- *C{25,26,27} is of the form $\left[\begin{array}{ccc} 0 & 0 & 1\\ 0 & 0 & 1\\ e & f & 1-e-f \end{array}\right],$ which generates a 2-face triangle*
  *C{25,26,20,19} is of the form $\left[\begin{array}{ccc} 0 & 0 & 1\\ 1-c & 0 & c\\ e & 1-e & 0 \end{array}\right],$ which generates a 2-face square*
- *C{25,26,27,19,20,21} is of the form $\left[\begin{array}{ccc} 0 & 0 & 1\\ d & 0 & 1-d\\ e & f & 1-e-f \end{array}\right],$ which generates a 3-face triangular prism*
- *C{25,26,10,11,16,17,19,20} is of the form $\left[\begin{array}{ccc} 0 & a & 1-a\\ 1-c & 0 & c\\ e & 1-e & 0 \end{array}\right],$ which generates a 3-face cube*
- *C{19,20,21,22,23,24,25,26,27} is of the form $\left[\begin{array}{ccc} 0 & 0 & 1\\ d & 1-c-d & c\\ e & f & 1-e-f \end{array}\right],$ which generates a 9-vertex 4-face*
- *C{25,26,27,10,11,12,16,17,18,19,20,21} is of the form $\left[\begin{array}{ccc} 0 & a & 1-a\\ d & 0 & 1-d\\ e & f & 1-e-f \end{array}\right],$ which generates a 12-vertex 4-face*
- *C{10,11,12,13,14,15,16,17,18,19,20,21,22,23,24,25,26,27} is of the form*
  *$\left[\begin{array}{ccc} 0 & a & 1-a\\ d & 1-c-d & c\\ e & f & 1-e-f \end{array}\right]$, which generates an 18-vertex 5-face, or facet.*

These examples suggest how to count the *face vector*, the vector whose components count the number of faces $f_{i}$ of dimension *i*, and more: the numbers of vertices of each type of face and how many of each type:

**Proposition** **A2.**
*The faces of $M_{3}$ are as follows:*

- *0-faces or vertices: the sets of matrices in $M_{3}$ with 6 zero entries in common. These are singletons and there are $f_{0}=27$ vertices.*
- *1-faces, or edges: these are the sets of matrices in $M_{3}$ with 5 zero entries in common. These are line segments between adjacent vertices; there are $f_{1}=81$ edges.*
- *2-faces: these are the sets of matrices with 4 zero entries in common. There are two types of such faces:*
  *Triangles: three vertices will generate a triangular face if there is a single row in which they differ: the vertices of a triangular face have all four zeros in two of the rows. Each vertex is attached, via its cap, to three distinct triangular faces. There are 27 triangles, no two of which abut one another.*
  *Squares: these are generated by four vertices and have a single zero each in two rows, and the remaining row with two zeros. There are 81 squares, so $f_{2}=108$.*
  *Equivalently, any two vertices which differ in exactly two rows will lie on a unique square. Every vertex is on 12 squares.*
- *3-faces: these are the sets of matrices with 3 zero entries in common. There are two types of such faces:*
  *Triangular Prisms are generated by 6 vertices, and with their zeros in two of the rows; there are 54 triangular prisms.*
  *Cubes are generated by 8 vertices, with a single zero in each of the three rows; there are 27 cubes and $f_{3}=81$.*
- *4-faces, or ridges: there are two such types of face, with 2 zero matrix entries in common.*
  *9-vertex faces of matrices in $M_{3}$ with 2 zero entries, in the same row. There are 9 such faces.*
  *12-vertex faces of matrices with 2 zero entries, in two different rows. There are 27 such faces, so $f_{4}=36$.*
- *5-faces, or facets: the sets, generated by 18 vertices, of matrices with 1 zero entry in common. There are $f_{5}=9$ such faces.*

Note that, as with any convex polytope, each face is again a polytope, whose faces are, in turn, themselves lower dimensional faces of $M_{3}$. For example, as in Figure A3, we can see that the 6 vertex 3-face is generated by two triangular faces “translated” along three square faces (or vice-versa), and the 8 vertex cube is a square translated along four square faces. The 9-vertex 4-face is a “quadra-triangular prism,” in that it consists of four triangular prisms wrapped together and the other 4-face has two triangular prisms joined by a cube. Similarly, the 5-facet is an extension of some of the previous faces along some others.

Note also that our 6-dimensional polytope $M_{3}$ embedded in $R^{d}$ with d=9 satisfies, as it should, the *Euler–Poincaré relation*

(A3) $$f_{0}-f_{1}+\ldots {\left(-1\right)}^{d-1}f_{d-1}=1+{\left(-1\right)}^{d}$$

With a view towards understanding the Markovian asymptotics, we now identify certain distinguished subsets of $M_{n}$.

**The Birkhoff Polytope and Periodic Sets**. An n×n matrix *Q* is *doubly stochastic* if all entries are non-negative and each row and each column sums to 1. The subset of all doubly stochastic matrices constitutes the *Birkhoff polytope* $B_{n}$. This is the sub-polytope of $M_{n}$ generated by a subset of n! of the $n^{n}$ vertices of $M_{n}$: 2 for $M_{2}$ and 6 for $M_{3}$. The vertices of the Birkhoff polytope are, under matrix multiplication, isomorphic to the symmetric group $S_{n}$ of permutations on the set $\bar{n}$ of *n* objects. For example, in the standard correspondence, the transposition $\left(13\right)\in S_{3}$, which exchanges objects 1 and 3, corresponds to the matrix $\left[\begin{array}{ccc} 0 & 0 & 1\\ 0 & 1 & 0\\ 1 & 0 & 0 \end{array}\right]$.

Since the vertices of $B_{n}$ form a finite group, each of its elements is cyclic. As transition matrices for Markov chains, then, the vertex matrices of $B_{n}$ are *periodic*: an n×n matrix *Q* being periodic if, for some integer *k*, $Q^{k}=I$, the least such *k* being the *period* of *Q*. The *periodic set* $P_{n}$ consists of all periodic stationary stochastic n×n matrices, and the *k*-*periodic set* $P_{n}^{k}$ consists of the period *k* matrices in P. For all *n*, there is only one period 1 matrix: $P_{n}^{1}=\left\{I\right\}$. Define a *derangement* in $S_{n}$, to be a permutation leaving no object of $\bar{n}$ in its place. Then, $P_{n}^{n}$ corresponds to the set of derangements in $S_{n}$.

In $M_{2}$, there is only one derangement matrix: the “NOT” operator $\left[\begin{array}{cc} 0 & 1\\ 1 & 0 \end{array}\right]\in P_{2}^{2}$ and $B_{2}$ is the line segment between NOT and the identity matrix (as in Figure A1).

In $M_{3}$, there are two vertices in $P_{3}^{3}$: the ones corresponding to the two derangements in $S_{3}$. There are three other vertices in $P_{3}^{2}$, which we call the *mirror* matrices. Together with the identity, we have identified all six vertices of $B_{3}$, numbered as in Figure A2:

$$\mathrm{Vertices of}\;B_{3}$$

(A4) $$P_{3}^{3}:\;\mathrm{EVEN}\;16:\left[\begin{array}{ccc} 0 & 1 & 0\\ 0 & 0 & 1\\ 1 & 0 & 0 \end{array}\right],\;\mathrm{ODD}\;20:\left[\begin{array}{ccc} 0 & 0 & 1\\ 1 & 0 & 0\\ 0 & 1 & 0 \end{array}\right]$$

(A5) $$P_{3}^{2}:\;\mathrm{Mirrors}\;\;22:\left[\begin{array}{ccc} 0 & 0 & 1\\ 0 & 1 & 0\\ 1 & 0 & 0 \end{array}\right],\;12:\left[\begin{array}{ccc} 0 & 1 & 0\\ 1 & 0 & 0\\ 0 & 0 & 1 \end{array}\right],\;8:\left[\begin{array}{ccc} 1 & 0 & 0\\ 0 & 0 & 1\\ 0 & 1 & 0 \end{array}\right]$$

(A6) $$P_{3}^{1}:\;\mathrm{Identity}\;\;6:\left[\begin{array}{ccc} 1 & 0 & 0\\ 0 & 1 & 0\\ 0 & 0 & 1 \end{array}\right]$$

Periodic matrices, being invertible, are of full rank and therefore every periodic *vertex* is in $B_{n}$. Periodicity in $M_{2}$ and $M_{3}$ only happens at vertices: no convex combination of periodic stochastic matrices can be periodic. We conjecture that this is generally true for all *n*:

**Conjecture** **A1.**
*$P_{n}$ is the vertex set of $B_{n}$, the set of all periodic stochastic matrices: $P_{n}^{n}$ corresponds to the set of n-cyclic derangements. For k=1,…,n, $P_{n}^{k}$ consists of all Birkhoff matrices corresponding to permutations $s\in S_{n}$ of the following kind: let $s=c_{1}c_{2}\ldots c_{l}$, where the $c_{i}$ are, up to order, the unique decomposition of s into its cycles. Let $k_{s}$ be the l.c.m. of the lengths of the cycles in s. Then, $P_{n}^{k}$ consists of those Birkhoff matrices corresponding to permutations $s\in S_{n}$, such that $k_{s}=k$ (For n=2,3 this comports with the fact that the number of derangements of k things is $!k=\left[k!/e\right]$, where e is the base of the natural logarithm and $\left[x\right]$ is the integer nearest to x).*

**The Stationary Complex and Fusion Complex.** We will refer to an idempotent stochastic matrix *A*, i.e., one satisfying $A^{2}=A$, as *stationary* (The term “stationary” refers to the fact that a Markov chain with such a transition matrix *A* is stationary: if the chain has an initial state given by μ, a measure on its state space, subsequent states are constant and equal to μA). The *stationary complex* $S_{n}$ consists of all stationary matrices in $M_{n}$. The next proposition says that the rank 1 matrices in $M_{n}$ constitute a polytope in $S_{n}$: this is the *fusion polytope* $F_{n}^{1}$. When n=2 this, together with the identity, fills up the stationary manifold: $S_{2}=F_{2}^{1}\cup \left\{I\right\}$ (see Figure A1). Its intersection with the Birkhoff polytope is a singleton consisting of the matrix each of whose rows is its stationary distribution

In $M_{3}$, the fusion polytope $F_{3}^{1}$ is shown in Proposition A3 below to be a 2-simplex. Except for its vertices, this polytope lies entirely in the interior of $M_{3}$, and its intersection with the Birkhoff polytope again consists of one matrix, each of whose rows is its stationary distribution $\left[\begin{array}{ccc} 1/3 & 1/3 & 1/3 \end{array}\right]$. There are now additional stationary matrices, namely the *partial fusion complex* $F_{n}^{2}$ which consists of the rank 2 matrices in the stationary manifold $S_{3}$. The collection of stationary stochastic matrices is the union of these disjoint sets: $S_{3}=F_{3}^{1}\cup F_{3}^{2}\cup \left\{I\right\}$. This is depicted in Figure A4, where solid lines are (boundary) edges of $M_{3}$ and dotted lines are in the interior (the numbering of vertices is as in Figure A2).

The figure shows it as a 1-dimensional curve, made up of 6 segments between its vertices. Moreover, it is connected in paired edges to the (full) fusion polytope $F_{3}^{1}$, a 2-dimensional simplex. Note, as in Proposition A2, the three triangular faces issuing from the identity. Additionally, 3 of the 12 square faces involving the identity actually connect the identity, as shown in the figure, to the full fusion manifold.

**Proposition** **A3.**

(i) *The fusion polytope $F_{n}^{1}$ is an (n−1)-dimensional simplex: the convex hull of the n vertex matrices $\left\{e_{i}\right\}$, where $e_{i}$ has all 1’s in the ith column and 0’s everywhere else. Other than its vertices, the fusion polytope lies entirely in the interior of $M_{n}$.*
(ii) *The vertices of the partial fusion complex $F_{3}^{2}$ constitute the cap of the identity and $F_{3}^{2}$ is the boundary of the hexagon made up of 6 linear segments between these (three of which are edges of $M_{3}$ and three are internal to it). $F_{3}^{2}$ has exactly three intersections with the Birkhoff polytope, each lying (as they should) in the middle of the interior segments joining the pairs of vertices (3,15), (5,9), and (4,24), respectively.*

Similarly, for k=2,…,n−1, the stationary matrices of rank *k* constitute the *rank k partial fusion complex* $F_{n}^{k}$ (We are not including in the fusion complex $F_{n}^{n}$, which would be just {I} and would involve *no* fusions).

We conjecture that the following facts, true for n= 2 and 3, are generally true for all *n*.

**Conjecture** **A2.**

(i) *The vertices of $F_{n}^{k}$, for k=1,…,n−1, consists of stochastic matrices of the following form: k columns, say $j_{1},\ldots ,j_{k}$ have a single 1 in their diagonal entries. One of these columns, say column $j_{1}$, has zeroes in the rows corresponding to the other diagonal 1’s (i.e., in the $j_{i}$th rows, i≠1) and 1’s otherwise; and the remaining columns of the matrix are all zeroes. There are, therefore, knk partial fusion vertices in $F_{n}^{k}$.*
(ii) *$F_{n}^{n-1}$ is the boundary of a figure made up of n(n−1) simplices of dimension n−2, whose vertices constitute the cap of the identity*

It is straightforward to see that the type of matrix in Conjecture A2(i) is indeed idempotent and of rank *k*. The conjecture will be proved upon showing that these are the *only* such stochastic matrices. The structure of the intermediate $F_{n}^{k}$ requires further study.

In identifying the Birkhoff polytope and stationary complex of $M_{3}$, we have exhausted 15 of its 27 vertices. The remaining 12 vertices are the members of the caps of derangement vertices 16 and 20, as in Figure A2. There, these two caps are just the columns of 6 vertices next to each of these two cyclic derangements, on the left and right of the figure, respectively. We will see later that it is useful to distinguish two further subsets each in each cap. With “E” for 16 and “O” for 20, these are:

(A7) Capof16E1:{10,17,21}E2:{7,13,18}

(A8) Capof20O1:{11,19,26}O2:{2,21,23}

### Appendix A.2. Dynamics in Markov Polytopes

We wish to study the flows of Markov chains on finite state spaces [89]; $M_{n}$ is the set of their transition kernels. Such a Markov chain is specified by giving both the kernel and a *stochastic vector* as its starting probability distribution. This is a 1×n matrix $\mu =(\mu_{1},\ldots ,\mu_{n})$, such that $0\leq \mu_{i}\leq 1$, for all *i* and $\sum_{i=1}^{n}\mu_{i}=1$. The stochastic vector μ is a *stationary distribution* for the stochastic matrix *Q* if μQ=μ.

**Lemma** **A1.**
*Every stochastic n×n matrix Q has a stationary distribution.*

In general, there may be more than one stationary distribution. However, if *Q* is irreducible and aperiodic, the stationary measure is unique.

By applying μQ=μ to any matrix in the **fusion polytope** $F_{n}^{1}$ we see, as in Proposition A3(i), that each row of *Q* is the same as μ. So the fusion polytope has unique stationary distributions. For a matrix in the partial fusion complex $F_{3}^{2}$, however, one coordinate of the stationary distribution is fixed at 0 (This is the coordinate corresponding to the column with one diagonal 1 and otherwise 0 s). Thus, the set of stationary distributions for a partial fusion matrix form a 1-simplex.

**Conjecture** **A3.**
*The sets of stationary distributions of matrices in the partial fusion complexes $F_{n}^{k}\;\left(k=1,\ldots ,n-1\right)$, are simplices of dimension n−k.*

First, we establish Figure A1 as representing the dynamics in $M_{2}$. Let $Q\left(x,y\right)=\left[\begin{array}{cc} 1-x & x\\ y & 1-y \end{array}\right]$ be the 2×2-stochastic-matrix-valued function on the parameter space, the unit square 0≤x,y≤1. Then a 2×1 matrix $\mu =\left[\begin{array}{cc} a & b \end{array}\right]$ is a stochastic vector if 0≤a,b,≤1,a+b=1. The following applies Proposition A3 to n=2: here the fusion complex is just the fusion polytope:

**Lemma** **A2.**
*If $Q\in M_{2}$ is stationary, then either:*

(i) *Q is in the fusion polytope and has equal rows: it is rank 1 and each row of Q is the unique stationary vector for Q, or*
(ii) *Q=I and every stochastic vector is stationary for Q.*

**Theorem** **A1.**

(i) *The fusion polytope $F_{2}^{1}$ corresponds to the segment {(x,y)|x,y∈[0,1];x+y=1}, including end-points.*
(ii) *The Birkhoff polytope $B_{2}$ corresponds to the segment x=y, including end-points. Its intersection with the fusion polytope is the singleton $\left\{\left[\begin{array}{cc} 1/2 & 1/2 \end{array}\right]\right\}$.*
(iii) *The periodic set $P_{2}^{2}$ corresponds to the green upper right corner Q(1,1). Its powers do not converge, but its stationary measure is $\left[\begin{array}{cc} 1/2 & 1/2 \end{array}\right]\}$.*
(iv) *The powers of any matrix other than the identity and the periodic point converge to the fusion complex, monotonically along the line between itself and the identity. In particular, the powers of Q(x,y), for (x,y)≠(0,0) and (x,y)≠(1,1), converge to the stationary matrix given by*
  (A9)
  $$\lim_{n\to \infty }Q{\left(x,y\right)}^{n}=Q\left(\frac{x}{x+y},\frac{y}{x+y}\right)=\left[\begin{array}{cc} \frac{y}{x+y} & \frac{x}{x+y}\\ \frac{y}{x+y} & \frac{x}{x+y} \end{array}\right]$$

We would like to study what aspects of the patterns we see in $M_{2}$ persist at higher dimensions and what new kinds of behavior emerge. Here, we make a bare start at this study. Consider Figure A5, which indicates some of the dynamics in $M_{3}$ where, for simplicity, we have indicated no adjacencies between the identity and its cap $F_{3}^{2}$, nor those between $F_{3}^{2}$ and $F_{3}^{1}$. The assertions made in Figure A5 are easily checked:

**Theorem** **A2.**
*Dynamics between the caps and the fusion polytope are as in Figure A5.*

### Appendix A.3. Proofs

**Proof of Proposition** **A1.** Suppose two vertices differ in the position of a unit entry in some row. Then it is straightforward to see a given convex combination of these two cannot be also a convex combination of any other pair of vertices. Thus, their convex hull is a 1-face, or edge, of the Markov polytope. Conversely, suppose two vertices $V_{1},V_{2}$ differ in two rows. WLOG, we can take these to be rows 1 and 2. Let the *j*th row of $V_{i},i,\;j=1,2$ be the 1×3 row vector $r_{ij}$ and assume $r_{11}\neq r_{21}$ and $r_{12}\neq r_{22}$. Let $V_{3}$ be the matrix equal to $V_{1}$, except in its *first* row and let $V_{4}$ be the matrix equal to $V_{1}$, except in its *second* row (an example is in Equations (A2) and (A3) above). Let *V* be the convex combination $V=xV_{1}+\left(1-x\right)V_{2}$, with x≥1/2. Then, *V* is *also* in the convex hull of $V_{1}$, $V_{3}$, and $V_{4}$: solving for $V=x^{\prime }V_{1}+y^{\prime }V_{3}+\left(1-x^{\prime }-y^{\prime }\right)V_{2}$, we get $y^{\prime }=1-x$ and $x^{\prime }=2x-1$. Our assertion will be verified if we can show that $0\leq x^{\prime },y^{\prime }\leq 1$. However, $x\geq 1/2\Rightarrow x^{\prime }\geq y\geq 0$ and $x\leq 1\Rightarrow x^{\prime }\leq 1$. A similar consideration applies to the instance x≤1/2, in which case we can see that *V* is now also a convex combination of $V_{2}$, $V_{3}$, and $V_{4}$. In either case, *V* is not on an edge. □

**Proof of Proposition** **A2.** (added new line before bullet)

- *0-faces* or *vertices*: a matrix in $M_{3}$ has 6 zero entries if it has three unit rows, each with three possibilities for its unit entry: there are 27 vertices.
- *1-faces*, or *edges*: the 5 zero entries can be distributed by choosing a row with a single zero in 3 ways, choosing that zero entry’s position in 3 ways, and then choosing places in the two other rows for their unit entry in 3 ways each: there are 81 edges.
- *2-faces*: one way a set of vertices can have 4 zero entries in common is if they all have two fixed zeros each of two rows (3 ways to choose this pair of rows). The zero placement, equivalently the unit entry, in each of these rows can be chosen in 3 ways each. Thus, there are $3^{3}=27$ triangular faces.
  Once this choice is made, the set generates a *Triangular face*, as the unit entry in the remaining row can be chosen in exactly three ways and the resulting vertices are all adjacent, by Proposition A1. Each vertex is adjacent to three distinct triangular faces, one for each row where the unit entry is shifted. Since there are three such distinguished rows, there are three distinct triangles including that vertex. Thus, no two triangles can abut one another (Another way to see that there are 81 triangles is to note that each of the 27 vertices is on 3 triangles, and all triangles are distinct).
  *Squares*: the other way a set of vertices will 4 zero entries in common is if they all have two rows (32=3 ways to choose this pair of rows) with a single zero each (3 ways to position the zero in each row), and the remaining row with two zeros (3 ways to position these zeros). There are, thus, 81 such faces.
  Each of the square faces will be the convex hull of the four vertices that are obtained as follows: call one of the vertices $V_{1}$ and let $V_{2}$ be a vertex which differs from $V_{1}$ in *both* rows. Pick one of the distinguished rows with a single zero and let $V_{3}$ be the vertex obtained by shifting $V_{1}$’s unit entry in that row, to its position in $V_{2}$. Let $V_{4}$ be the vertex obtained from $V_{1}$ by shifting its unit entry in the *other* distinguished row to that of $V_{2}$. There are, by Proposition A1, edges between $V_{1}$ and $V_{3}$, $V_{1}$ and $V_{4}$, $V_{3}$ and $V_{2}$, and $V_{4}$ and $V_{2}$ and no other edges: the resulting figure, which (see Example A1) has two free parameters, is indeed a 2-dimensional square.
  Given an arbitrary vertex, there are 3 ways to choose two of its rows, and 4 ways to change the position of one or both unit entries in those two rows. Thus, any given vertex will lie on 12 squares.
- *3-faces*: sets of vertices with 3 zero entries in common can happen in two ways:
  Their common 3 zeros lie in two of the rows: one with 2 zeros and the other with one. Pick the 2-zero row in 3 ways and position the 2 zeros in three ways. Then, pick the 1-zero row in two ways and the position of the zero in three ways; there are 54 such faces.
  Such a set will have 6 vertices, as there are two possible positions for the unit entry in the single-zero row, and three in the row with no shared zeros. Example 1 shows how to see that the resulting figure generated by these 6 vertices is a *Triangular Prism*, as in Figure A3.
  Similarly, *cubes* are generated by 8 vertices, with a single zero in each of the three rows; there are 3×3×3=27 cubes.
- *4-faces*, or *ridges* have 2 zero entries in common: when these are in the *same* row, the remaining two rows are free, so there are 3×3=9 vertices in such a face. Pick the row with 2 zero entries in common in 3 ways, and place those zeros in 3 ways: there are 9 such faces.
  When the 2 zero entries in common are in two *different* rows, we get a *12 vertex face*, since there are 2×2×3=12 ways to distribute unit entries in the rows. Pick the two distinguished rows in 3 ways and distribute the two common zeros in 3 ways each. There are, thus, 27 such faces.
- The *5-faces*, or *facets* are those 18-vertex faces (18=2×3×3) with 1 zero entry in common. There are 3×3=9 such faces.

□

**Proof of Proposition** **A3.**

(i) A rank 1 stochastic matrix *Q* is one whose rows are all the same stationary distribution, say $\mu =(\mu_{1},\ldots ,\mu_{n})$. Thus $Q=\sum_{i=1}^{n}\mu_{i}e_{i}$. Since each row of *Q* is equal to μ and its *j*th column is $\mu_{j}1$, where 1 is the column of all 1’s, we obtain
  $${\left(Q^{2}\right)}_{ij}=\mu \cdot \left(\mu_{j}1\right)=\mu_{j}\sum_{i=1}^{n}\mu_{i}=\mu_{j}$$
  In other words, $Q^{2}=Q$: rank 1 stochastic matrices are idempotent and so constitute $F_{n}^{1}$.
(ii) It can be verified from Figure A2 that $F_{3}^{2}$, the idempotent rank 2 vertices, constitute the cap of the identity and that it is the boundary of the hexagon made up of 6 linear segments between these (three of which are edges of $M_{3}$ and three are internal to it) (A quick way to verify that any segment between idempotent matrices A,B is itself idempotent is to prove that this is so if their anticommutator {A,B}:=AB+BA is equal to A+B).

□

**Proof of Lemma** **A1** *Q* can be viewed as a linear (and so continuous) operator on $R^{n}$, which restricts to a continuous function from the convex set of probability measures on $\bar{n}:=\left\{1,\ldots ,n\right\}$ to itself. Brower’s fixed point theorem then says that there is a fixed point of the continuous map *Q* of the convex set of probability measures on $\bar{n}:=\left\{1,\ldots ,n\right\}$ to itself: this fixed point is what is meant by a stationary distribution. □

**Proof of Theorem** **A1** Items (i), (ii), and (iii) are straightforward. Recall that the stochastic matrix with parameters x,y is given by

$$Q\left(x,y\right)=\left[\begin{array}{cc} 1-x & x\\ y & 1-y \end{array}\right]$$

with x,y∈[0,1].

(iv) Fix $p_{0}=\left(x_{0},y_{0}\right)$ in the unit square, except for the corners (0,0) and (1,1). Points on the line between the origin and $p_{0}$ can be parameterized as $p_{a}=\left(ax_{0},ay_{0}\right)$. A calculation shows that
  $$Q\left(p_{a}\right)Q\left(p_{0}\right)=Q\left(x_{0}\left(1+a-a\left(x_{0}+y_{0}\right)\right),y_{0}\left(1+a-a\left(x_{0}+y_{0}\right)\right)\right)$$
  Thus $Q\left(p_{a}\right)Q\left(p_{0}\right)=Q\left(p_{1}\right)$, where $p_{1}=\left(x_{1},y_{1}\right)=\left(\left(1+a-a\left(x_{0}+y_{0}\right)\right)x_{0},\left(1+a-a\left(x_{0}+y_{0}\right)\right)y_{0}\right)$ is again a point on that same line. Iterating the above formula starting at $p_{0}$, we see that the sequence ${\left(Q{\left(p_{0}\right)}^{n}\right)}_{n=1}^{\infty }$ stays on that line. Suppose $p_{0}$ is to the right of the cross-diagonal x+y=1. In terms of our parameter the cross-diagonal is intersected at $a=\frac{1}{x_{0}+y_{0}}$. This means that if $a>\frac{1}{x_{0}+y_{0}}$, our formula shows that $x_{1}<ax_{0}$ and $y_{1}<ay_{0}$: $p_{1}$ is shifted from $p_{0}$ a positive distance *towards* the origin. Similarly, when $p_{0}$ is to the left of x+y=1, so that $a<\frac{1}{x_{0}+y_{0}}$, then $p_{1}$ is shifted *away* from the origin. In either case, the sequence ${\left(Q{\left(p_{0}\right)}^{n}\right)}_{n=1}^{\infty }$ is *strictly* monotonic towards the cross-diagonal, unless it is actually on it, where the matrix Q(x,y) is stationary.

□

## Appendix B. Decorated Permutations for the 27 Vertices of the Markov Polytope $M_{3}$

**Number**	**Matrix**	**Decorated Permutation**
1	$\left[\begin{array}{ccc} 1 & 0 & 0\\ 1 & 0 & 0\\ 1 & 0 & 0 \end{array}\right]$	[4,2,3]
2	$\left[\begin{array}{ccc} 1 & 0 & 0\\ 1 & 0 & 0\\ 0 & 1 & 0 \end{array}\right]$	[4,2,3]
3	$\left[\begin{array}{ccc} 1 & 0 & 0\\ 1 & 0 & 0\\ 0 & 0 & 1 \end{array}\right]$	[4,2,6]
4	$\left[\begin{array}{ccc} 1 & 0 & 0\\ 0 & 1 & 0\\ 1 & 0 & 0 \end{array}\right]$	[4,5,3]
5	$\left[\begin{array}{ccc} 1 & 0 & 0\\ 0 & 1 & 0\\ 0 & 1 & 0 \end{array}\right]$	[4,5,3]
6	$\left[\begin{array}{ccc} 1 & 0 & 0\\ 0 & 1 & 0\\ 0 & 0 & 1 \end{array}\right]$	[4,5,6]
7	$\left[\begin{array}{ccc} 1 & 0 & 0\\ 0 & 0 & 1\\ 1 & 0 & 0 \end{array}\right]$	[4,2,3]
8	$\left[\begin{array}{ccc} 1 & 0 & 0\\ 0 & 0 & 1\\ 0 & 1 & 0 \end{array}\right]$	[4,3,5]
9	$\left[\begin{array}{ccc} 1 & 0 & 0\\ 0 & 0 & 1\\ 0 & 0 & 1 \end{array}\right]$	[4,2,6]
10	$\left[\begin{array}{ccc} 0 & 1 & 0\\ 1 & 0 & 0\\ 1 & 0 & 0 \end{array}\right]$	[2,4,3]
11	$\left[\begin{array}{ccc} 0 & 1 & 0\\ 1 & 0 & 0\\ 0 & 1 & 0 \end{array}\right]$	[2,4,3]
12	$\left[\begin{array}{ccc} 0 & 1 & 0\\ 1 & 0 & 0\\ 0 & 0 & 1 \end{array}\right]$	[2,4,6]
13	$\left[\begin{array}{ccc} 0 & 1 & 0\\ 0 & 1 & 0\\ 1 & 0 & 0 \end{array}\right]$	[1,5,3]
14	$\left[\begin{array}{ccc} 0 & 1 & 0\\ 0 & 1 & 0\\ 0 & 1 & 0 \end{array}\right]$	[1,5,3]
15	$\left[\begin{array}{ccc} 0 & 1 & 0\\ 0 & 1 & 0\\ 0 & 0 & 1 \end{array}\right]$	[1,5,6]
16	$\left[\begin{array}{ccc} 0 & 1 & 0\\ 0 & 0 & 1\\ 1 & 0 & 0 \end{array}\right]$	[3,4,5]
17	$\left[\begin{array}{ccc} 0 & 1 & 0\\ 0 & 0 & 1\\ 0 & 1 & 0 \end{array}\right]$	[1,3,5]
18	$\left[\begin{array}{ccc} 0 & 1 & 0\\ 0 & 0 & 1\\ 0 & 0 & 1 \end{array}\right]$	[1,2,6]
19	$\left[\begin{array}{ccc} 0 & 0 & 1\\ 1 & 0 & 0\\ 1 & 0 & 0 \end{array}\right]$	[3,2,4]
20	$\left[\begin{array}{ccc} 0 & 0 & 1\\ 1 & 0 & 0\\ 0 & 1 & 0 \end{array}\right]$	[3,4,5]
21	$\left[\begin{array}{ccc} 0 & 0 & 1\\ 1 & 0 & 0\\ 0 & 0 & 1 \end{array}\right]$	[1,2,6]
22	$\left[\begin{array}{ccc} 0 & 0 & 1\\ 0 & 1 & 0\\ 1 & 0 & 0 \end{array}\right]$	[3,5,4]
23	$\left[\begin{array}{ccc} 0 & 0 & 1\\ 0 & 1 & 0\\ 0 & 1 & 0 \end{array}\right]$	[1,5,3]
24	$\left[\begin{array}{ccc} 0 & 0 & 1\\ 0 & 1 & 0\\ 0 & 0 & 1 \end{array}\right]$	[1,5,6]
25	$\left[\begin{array}{ccc} 0 & 0 & 1\\ 0 & 0 & 1\\ 1 & 0 & 0 \end{array}\right]$	[3,2,4]
26	$\left[\begin{array}{ccc} 0 & 0 & 1\\ 0 & 0 & 1\\ 0 & 1 & 0 \end{array}\right]$	[1,3,5]
27	$\left[\begin{array}{ccc} 0 & 0 & 1\\ 0 & 0 & 1\\ 0 & 0 & 1 \end{array}\right]$	[1,2,6]
